# Molecular pathways and therapeutic targets in lung cancer

**DOI:** 10.18632/oncotarget.1891

**Published:** 2014-04-08

**Authors:** Emma Shtivelman, Thomas Hensing, George R. Simon, Phillip A. Dennis, Gregory A. Otterson, Raphael Bueno, Ravi Salgia

**Affiliations:** ^1^ Cancer Commons, Palo Alto, CA, United States of America; ^2^ Northshore University Health System and University of Chicago, United States of America; ^3^ MD Anderson Cancer Center, United States of America; ^4^ Johns Hopkins University School of Medicine, United States of America; ^5^ Ohio State University Comprehensive Cancer Center, United States of America; ^6^ Brigham & Women's Hospital, Harvard Medical School, United States of America; ^7^ Department of Medicine, Section of Hematology/Oncology, University of Chicago, United States of America

**Keywords:** lung cancer, targeted therapy, immune therapy

## Abstract

Lung cancer is still the leading cause of cancer death worldwide. Both histologically and molecularly lung cancer is heterogeneous. This review summarizes the current knowledge of the pathways involved in the various types of lung cancer with an emphasis on the clinical implications of the increasing number of actionable molecular targets. It describes the major pathways and molecular alterations implicated in the development and progression of non-small cell lung cancer (adenocarcinoma and squamous cancer), and of small cell carcinoma, emphasizing the molecular alterations comprising the specific blueprints in each group. The approved and investigational targeted therapies as well as the immune therapies, and clinical trials exploring the variety of targeted approaches to treatment of lung cancer are the main focus of this review.

## TABLE OF CONTENTS

INTRODUCTION
1. MOLECULAR TARGETS IN LUNG CANCER
1.1 RECEPTOR TYROSINE KINASES (RTKs)
1.2. RAS PATHWAY
1.3. BRAF/MAPK PATWHAY
1.4. PI3K PATHWAY
1.5. LKB1 (STK11)/AMPK PATHWAY
1.6. TP53 PATHWAY
1.7. RB1 PATHWAY
1.8. MYC PATHWAY
1.9. EPIGENETIC PATHWAYS
1.10. OXIDATIVE STRESS
1.11. DEVELOPMENTAL PATHWAYS
1.12. OTHER RECURRENT ALTERATIONS
2. IMMUNOTHERAPY FOR LUNG CANCER
2.1. Blockade of immune checkpoints
2.2 Vaccine interventions
CONCLUDING REMARKS
ACKNOWLEDGEMENTS
REFERENCES

## INTRODUCTION

Lung cancer is the leading cause of cancer death worldwide. Approximately 80% of lung cancers are grouped as non-small cell lung carcinoma (NSCLC), which are clinically and pathologically different from small cell lung carcinoma (SCLC). Adenocarcinoma (AC) of lung comprises about 40% to 50% of all lung cancers. AC is the most common subtype of NSCLC, the group that also includes squamous cell carcinoma (SQCC) and large cell carcinoma (LCLC). Recently, genomic-based classification of LCLC eliminated this subtype, demonstrating that it can be molecularly sub-classified as either adenocarcinoma or neuroendocrine tumor [1]. The discovery of recurrent mutations in EGFR and fusions involving ALK and other receptor tyrosine kinases (RTKs), and development of targeted therapies, have transformed the standard of treatment of patients with AC. Molecular genotyping of AC now routinely includes testing the status of EGFR and ALK, which are altered in about 25% of AC patients, and for which there are approved targeted therapies. Patients with alterations in EGFR and ALK respond better to targeted inhibitors than to conventional chemotherapy.

SQCC tumors rarely have mutations in EGFR or ALK fusions, instead harboring alterations in other RTKs – mutations in DDR2 and amplifications/mutations of FGFR (fibroblast growth factor receptor) family, both of which are therapeutic targets. Analysis of signaling pathways involved in pathogenesis and large-scale genomic analyses of SQCC reveal it to be a distinct clinicopathological type of NSCLC, even though some somatogenic alterations are shared with AC (Tables 1 and 2). SCLC, however, is an entirely distinct type of lung cancer, originating in a different cellular compartment of lung tissue, and with its own distinct molecular pathogenesis as well as pathology and clinical course.

**Table 1:** CELLULAR PATHWAYS WHOSE ACTIVITY IS AFFECTED BY SOMATIC ALTERATIONS IN LUNG CANCERS *

PATHWAY	AC		SQCC		SCLC	
	%	Genes involved	%	Genes involved	%	Genes involved
RTK	50%	EGFR, ALK, MET, ERBB2, ROS, RET	27%	EGFR, FGFR1-3, ERBB2, ERBB3, DDR2	6%	FGFR1
RAS/RAF	25%	KRAS, NF1, BRAF, NRAS	22%	NF1, KRAS, HRAS, NRAS, RASA1, BRAF		
PI3K/AKT	10-12%	PIK3CA, PTEN, AKT1	59%	PIK3CA, PTEN, AKT1, AKT2, AKT3, TSC1-2	10%	PTEN
LKB1/AMPK	15-30%	LKB1				
TP53	50%	P53, MDM2	80%	TP53	80-90%	TP53
RB1/ CDKNA2	15-20	CDKNA2	79%	CDKNA2, RB1	100%	RB1, CCNE1
MYC	30%	MYC			16-30%	MYC, MYCN, MYCL
Epigenetic regulation	22%	SMARCA4, ARID1A, SETD2	20%	MLL2	19%	EP300, CREBBP, MLL
Developmental pathway	20%	NKX2.1/TTF1	44%	SOX2, TP63, NOTCH1, NOTCH2, ASCL4, FOXP1	20%	SLIT2, EPHA7
Oxidative stress response	10%	KEAP1	34%	KEAP1, NRF2, CUL3		

* The genes involved in each type of lung cancer are listed in order of the frequency of alterations found

**Table 2:** MAJOR SOMATIC ALTERATIONS IN LUNG CANCER

Gene	PATHWAY	ABERRATION	%AC	%SCC	%SCLC	Drugs, approved and investigational
EGFR	RTK	Mutation Amplification	20-30% >20%	rare 7%		Erlotinib, Gefitinib, Afatinib (approved), Dacomitinib, Cetuximab, Necitumumab, Neratinib
ALK		Fusion with EML4 and other rare partners	3-13%			Crizotinib (approved), X-396, LDK378; Ganetispib, AUY922, AT13387
MET		Mutation, amplification post-treatment with EGFR inhibitor	5% 20%			Tivantinib, Cabozantinib, Ornatuzumab, Tivantinib
ERBB2		Mutation, Amplification	2-4% 5-10%			Trastuzumab, Afatinib, Neratinib, MGHA22
ERBB3		Mutation		2%		MM-121
ROS		Mutation	1.5%			Crizotinib, AT13387 (HSP90)
RET		Translocation with KIF5B and other genes	1-2%			Vandetanib, Cabozantinib?
FGFR1		Amplification	1-3%	22%	6%	AZD4547, BGJ 398, BIBF 1120/nintedanib, dovitinib, HGS1036
DDR2		Mutation		3.8%		Dasatinib
IGF1R		Overexpression	ND	ND	95%	AXL1717, OSI-906
KRAS	RAS	Mutation	30%	5%		Selumetinib, Trametinib, MEK162, and BKM120, everolimus,sirolimus AUY922, BYL719,Reolysin MEK162
NF1			8-10%	11%		
HRAS				3%		
NRAS			<1%	<1%		
RASA1				4%		
BRAF	RAF	Mutations	6%	4%		Vemurafenib (only for V600E)
PIK3CA	PI3K	Mutation	rare	16%		BKM120, PX-866, GDC-0941
PTEN		Deletion	rare	8%		BKM120, PX-866, GDC-0941 (PI3K), MK-2206 (AKT)
AKT1,2,3			rare	16%(AKT3) 20% all		MK-2206
TSC1,2				6%		Everolimus, sirolimus, temsirolimus
LKB1	LKB1/AMPK	Mutation	15-30%	2%		Biguanide compounds
TP53	TP53	Mutation Copy number loss	50% 20%	80%	70%	
MDM2		Amplification	20%			Inhibitors of TP53 - MDM2 interaction
CDKNA2 /p16INK4	RB1/ CDK	Deletions, silencing, mutation	>20%	72%		CDK inhibitors PD0332991, BAY1000394
RB1		Mutation	rare	7%	100%	
CCNE1		Copy gain	12%			
MYC MYCN MYCL	Transcriptional regulators	Amplification	31%	rare	16%	Aurora kinase inhibitors, BH3 mimetics
SMARCA4	Epigenetic regulation	Mutation	10%			
ARID1A		Mutation	8%			
SETD2		Mutation	5%			
MLL2		Mutation		19%		
EP300		Mutation			9%	
CREBBP		Mutation			9%	
MLL		Mutation			10%	
KEAP1	Oxidative stress response	Mutation	11%	12%		
NRF2		Mutation		19%		
CUL3		Mutation		7%		
NKX2.1/TTF1	Develop-mental pathways	Amplification	20%			
SOX2		Amplification, overexpression		21%		
TP63		Amplification, overexpression		16%		
NOTCH1, NOTCH 2		Mutation		8 and 5%		
ASCL4		Mutation		3%		

In spite of advances in targeting signaling pathways in NSCLC, the most prevalent mutations found in lung cancer are those within the tumor suppressor TP53, which are currently untargetable. ACs often contain loss-of-function mutations in other tumor suppressor genes–the most prominent, in addition to TP53, being loss of function of LKB1/STK11, NF1, CDKN2A, SMARCA4, and KEAP1. SQCC tumors have a different and partially overlapping with AC spectrum of inactivated tumor suppressors: TP53 in a majority of tumors and inactivation of any of the following: CDKN2A, PTEN, KEAP1, MLL2, HLA-A, NFE2L2, NOTCH1 and RB1. These are challenging therapeutic targets, even though strategies for indirect targeting, i.e., involving proteins activated in the pathways governed by tumor suppressors, are being developed.

A number of targets other than EGFR and ALK are explored in clinical and preclinical development, and most of them are altered in AC in a gain-of-function manner, i.e., increase in enzymatic activity that could be targeted. Among these targets are KRAS, BRAF, ERBB2, PI3KC and translocations involving RET and ROS, as well as RTK MET, which is activated mainly in tumors that develop resistance to targeted therapies.

A large study employing next generation sequencing (NGS) to identify mutations and other alterations in AC was conducted in 2012, sequencing exomes or genomes of 183 ACs [2]. The mean exomic somatic mutation rate in this study was 12 per megabase, but there were large differences in numbers of mutations observed in tumors from non-smokers versus smokers: mean 2.9/Mb versus 12.9/Mb. In an earlier smaller study the difference was reported to be 10-fold [3]. The authors [2] noted that a significant number of driver mutations grouped with epigenetic or RNA deregulation, which has not been recognized in the pre-genomics era. These are interesting observations regarding the pathogenesis of AC but it is possible that they could be explained in part by the failure to identify all driver mutations.

A large NGS analysis of SQCC (178 tumors) performed by the Cancer Genome Atlas Research Network identified a mean of 360 exomic mutations, 323 altered copy number segments and 165 genomic rearrangements per tumor. The mean somatic mutation rate of 8.1 mutations per megabase observed in this study of SQCC was higher than that previously reported for a number of other types of cancer. The spectrum of somatic copy number alteration in SQCC was in general similar to AC with a notable exception of selective amplification of a region on chromosome 3q. The evidence of unique clinical and pathological character of SQCC was confirmed in this study [4] that identified SOX2 amplification, NFE2L2 and KEAP1 mutations, PI3K pathway changes, FGFR1 amplification, and DDR2 mutations that are not encountered or are relatively rare in AC.

Small cell lung cancer is almost exclusively associated with smoking, with about 90% of patients being smokers or former smokers. SCLC patients with extensive have a poor prognosis, even though the initial response to chemotherapy could be positive. Relapse usually occurs within months, and the recurrent disease is almost invariably lethal. Integrative genome analysis of 29 SCLC tumors [5] has determined an extremely high mutation rate of 7.4 protein-changing mutations per megabase. It also confirmed that all SCLC tumors have inactivated TP53 and RB pathways and recurrent amplifications of MYC genes, known to be a hallmark of SCLC prior to the evolution of NGS methodologies. In addition, the study found recurrent mutations in histone modifiers CREBBP, EP300, and MLL. Involvement of signaling pathways in SCLC is more limited than in other lung cancer types, with 6% of tumors carrying amplified FGFR1, and PTEN inactivation occurring in some tumors. Profound disregulation of cell cycle control involving the already mentioned alterations in TP53 and in RB1 and MYC genes is a ubiquitous feature of SCLC. Disregulation of the developmental pathways was noted in about 10% of tumors through mutations in SLIT2 and EPHA7. Proteomic profiling identified EZH2 and PARP-1 as activated and highly expressed proteins in SCLC [6].

This paper will review alterations in a number of cellular pathways involved in pathogenesis of lung cancer, emphasizing those that contain actionable targets, and providing information on clinical testing of relevant investigational drugs. Table 1 lists pathways and genes products known to be involved in three major types of lung cancer, and Table 2 contains a more detailed description of gene products and frequency of their alterations as well as potential drugs. It is obvious from Table 1 that RTK signal transduction pathways are predominantly affected in AC, and much less so in SQCC and particularly in SCLC, while the PI3K pathway is deregulated in more than half of SQCC. SCLC is distinguished by universal disruption of both TP53 and RB1 pathways, even though, as mentioned, TP53 itself is the most frequently mutated gene in all types of lung cancer. Interestingly, epigenetic deregulation is observed in all three types with similar frequency but involves different sets of genes that are mutated. Developmental or differentiation pathways are involved most prominently in SQCC and only one developmental gene is affected by recurrent mutations in AC.

### 1 Molecular targets in lung cancer

#### 1.1 RECEPTOR TYROSINE KINASES (RTKs)

RTKs are involved in different types of lung cancer at different frequencies, but have attracted a lot of interest because there are targeted therapies for activated RTKs approved or fairly advanced in clinical development. Testing for mutations in EGFR and ALK translocation in AC has become a routine diagnostic procedure, in line with NCCN guidelines, because treatment of EGFR mutant and ALK-positive AC with FDA-approved targeted drugs produces better responses than chemotherapy regimens.

##### EGFR

EGFR is a receptor tyrosine kinase that binds to epidermal growth factor and other growth factor ligands to become activated. Upon activation, EGFR tyrosine kinase activates downstream pathways including MAPK and PI3K, leading to DNA synthesis and cell proliferation. Activation of EGFR has other pleiotropic effects, among them contributing to immune escape of tumors [7] and to suppression of autophagy [8], both with serious clinical implications for immunotherapy approaches and the currently explored therapeutic induction of autophagy, respectively.

EGFR is implicated in a variety of cancers and frequently mutated in AC. EGFR alterations occur in ~10% of western and ~50% of Asian patients with lung AC. Higher EGFR mutation frequency is observed in non-smokers, women, and in non-mucinous tumors. EGFR has been shown to be deregulated by various mechanisms in AC, including overexpression, amplification, and mutation. Overexpression of EGFR occurs in up to 60% of NSCLC, with higher frequency in AC versus SQCC (reviewed in [9, 10]).

Several types of activating mutations are known to occur in EGFR in NSCLC: Class I - exon 19 in-frame deletions (44% of all EGFR mutations), Class II - single amino acid changes (L858R 41%, G719 4%, other missense mutations 6%), Class III - exon 20 in-frame duplication/insertions (5%). These mutations occur in the tyrosine kinase domain of EGFR. Eighty-five percent of all EGFR-activating mutations are exon 19 in-frame deletions or L858R, and they tend to be sensitive to currently approved EGFR inhibitors (reviewed in [11]). Class III mutations (exon 20) are generally insensitive to EGFR inhibitors with exception of A763_Y764insFQEA [12].

Patients with AC and “large cell” histology NSCLC should be tested at diagnosis for EGFR mutations, as those who exhibit such mutations benefit from EGFR inhibitors (e.g., erlotinib, gefitinib, or afatinib) in the first-line setting. Data from several clinical trials have indicated that patients with EGFR mutations have improved progression-free survival (PFS) and overall survival (OS) when treated with EGFR inhibitors compared to patients with the same mutations who received standard-of-care, cytotoxic chemotherapy regimens [13-15].

##### Potential therapeutic approaches for EGFR-mutant AC

EGFR inhibitors. Table 3 lists many of the EGFR inhibitors as well as other targeted drugs under development for treatment of NSCLC, and a comprehensive list of targeted therapies for NSCLC is regularly updated and published [16]. FDA-approved EGFR inhibitors include erlotinib (Tarceva), gefitinib (Iressa) and afatinib (Gilotrif). A randomized trial in Japan showed a significantly prolonged PFS in patients on gefitinib versus standard chemotherapy [17]. This trial was the first randomized study to demonstrate benefit of an oral targeted therapy versus chemotherapy in a molecularly selected population with NSCLC. However, FDA withdrew approval for use of gefitinib in new patients due to lack of evidence that it extends life. In May 2013 FDA approved a companion diagnostic test for erlotinib (Cobas) that detects exon 19 deletions or exon 21 L858R substitutions. These mutations are found in patients that are selectively good responders to erlotinib therapy. In a pivotal trial of erlotinib versus chemotherapy, PFS of 13.1 months was seen with erlotinib treatment, compared to 4.6 months for those who received a standard two-drug chemotherapy regimen [18].

**Table 3:** TARGETED DRUGS IN DEVELOPMENT OR APPROVED FOR LUNG CANCER

Drug	Target	Manufacturer
Erlotinib *	EGFR	Roche
PF-00299804/Dacomitinib	EGFR	Pfizer
Cetuximab/Erbitax **	EGFR (Mab)	Bristol-Myers Squibb
Gelfitinib/Iressa *	EGFR	AstraZeneka
Afatinib/BIB2992*	EGFR, ERBB2	Boehringer Ingelhime
CO-1686	EGFR T790M	Clovis
Icotinib	EGFR	Zhejiang Beta Pharma Inc
Necitumumab/ IMC-11F8	EGFR (Mab)	Lilly
Nimozutumab	EGFR (Mab)	Biocon
Neratinib	EGFR, ERBB2, ERBB4	Pfizer
MGHA22	ERBB2 (Mab)	Macrogenics
MM-121	ERBB3	Merrimack
AP2611	ALK, EGFR, ROS	ARIAD Pharmaceutical
ASP3026	ALK, ROS	Astellas Pharma
Crizotinib/Xalkori/PF-02341066 *	ALK, ROS	Pfizer
X-396	ALK	Xcovery
LDK378	ALK	Novartis
RO5424802/ CH5424802	ALK	Roche, Chugai Pharmaceutical
GSK1363089/XL880/Foretinib	MET, VEGFR	GlaxoSmithKlein
Cabozantinib (XL184) **	MET, RET	Exelixis
Onartuzumab/ MetMAb	MET (Mab)	Genentech
INC280	MET	Novartis/Incyte
ARQ 197/Tivantinib	MET	ArQule and Daiichi Sankyo
AMG-102	HGF (Mab)	Amgen
MGCD265	MET, VEGFRs, Axl, Tie2, Ron	MethylGene
AMG 102/Rilotumumab	HGF (MET ligand)	AMGEN
Linsitinib/OSI-906	IGF1-R	OSI Pharmaceuticals
Figitumumab	IGF1-R	Pfizer
cixutumumab	IGF1-R	Imclone
AMG 479	IGF1-R (Mab)	Amgen
OSI-906/linsitinib	IGF1-R	OSI Pharmaceuticals
AXL1717/ Picropodophyllin	IGF1-R	Axelar AB
Vandetanib/ZD6474	RET	AstraZeneka
BGJ398	FGFR	Novartis
Dovitinib/TKI258	FGFR	Novartis
AZD 4547	FGFR	AstraZeneka
HGS1036	FGFR	Five Prime Therapeutics
Tivozanib	VEGFRs	Aveo Oncology
Cediranib maleate/AZD2171/Recentin	VEGFR	AstraZeneca
BIBF 1120/ nintedanib	VEGFR, FGFR and PDGFR	Boehringer Ingelheim
HGS1036	FGFR	
Reolysin	Activated RAS, KRAS	Oncolytics Biotech
PX-866	PI3K	Oncothyreon
BKM120	PI3K	Novartis
GDC0941	PI3K	Genentech
BEZ235	PI3K/mTOR	Novartis
BYL719	PI3K/mTOR	Novartis
SAR245409	PI3K/mTOR	Sanofi
Sirolimus/Rapamune	mTOR	Pfizer
CCI-779/temsirolimus	mTOR	Pfizer
Everolimus/RAD-001	TOR	Novartis
DS-3078a	mTOR	Daiichi Sankyo
CC-223	TORC1, 2	Celgene
Temsirolimus	mTOR	Wyeth Pharmaceuticals
MK2206	AKT	Merck
GSK2141795	AKT	GlaxoSmithKlein
RAF265	RAF	Novartis
MSC1936369B	MEK	Merck
RO4987655	MEK	Hoffman-La-Roche
MEK162 trametinib/GSK1120212	MEK	GlaxoSmithKlein
GDC-0973/XL518	MEK	Exelixis
AZD6244/selumetinib	MEK	AstraZeneca
Visomodegib/GDC-0449	Hedgehog	Genentech
Vanctitumab/OMP18R5	FZD7	OncoMed Pharmaceuticals Inc
MLN8237/alisertib	Aurora kinase	Millenium
PD0332991	CDK4/6	Pfizer
BAY1000394	Pan CDK	Bayer
AT13387	HSP90	Astex Pharmaceuticals
AUY922	HSP90	Novartis
STA-9090 (Ganetespib)	HSP90	Synta Pharmaceuticals
Retaspimycin/IPI-504	HSP90	Infinity
Panobinostat/ LBH589	Histone deacetylases	Novartis
Entinostat/MS-275	Histone deacetylases	Syndax
BMN-673	PARP	BioMarin
Veliparib	PARP	Abbot
**Immunomodulatory antibodies**		
Ipilimumab/Yervoy **	CTLA-4, blocking antibody	Bristol-Myers Squibb
BMS-936558/MDX-1106/ONO-4538	PD-1, blocking antibody	Bristol-Myers Squibb
MK-3475/Lambrolizumab	PD-1, blocking antibody	Merck
BMS-936559	PD-L1, blocking antibody	Bristol-Myers Squibb
MPDL3280A/RG7446	PD-L1, blocking antibody	Roche
MEDI4736	PD-L1, blocking antibody	AstraZeneca

* FDA approved for NSCLC

** FDA approved for other malignancies

Icotinib is another EGFR inhibitor clinically developed in China and reportedly active against mutant and wild-type forms of the receptor. A phase 3 randomized trial of icotinib versus gefitinib showed the non-inferiority of icotinib and a much better profile in terms of drug-related side effects [19].

Second-generation EGFR inhibitors that bind to EGFR or other receptors in the EGFR family in an irreversible manner are in clinical testing (reviewed in [20]). BIBW2992/Afatinib is an irreversible ErbB family blocker that covalently binds to the cysteine residue of EGFR (as well as HER2), providing longer inhibition of EGFR (reviewed in [21]). Afatinib showed objective responses in 60% of molecularly selected patients in a phase 2 study, LUX-Lung 3 [22]. Afatinib has also demonstrated a small benefit in those previously treated with other EGFR TKIs [23]. In a decisive large randomized trial, afatinib in a preselected EGFR-mutant population was compared to the state-of-the-art chemotherapy with pemetrexed and cisplatin, and was shown to be superior [24]. Afatinib is undergoing clinical testing as single therapy or in combination treatments in at least 20 trials (for example, NCT01542437, NCT01466660). In July 2013 afatinib (Gilotrif) was approved as the first-line treatment for advanced stage NSCLC with class I and class II EGFR mutations.

Dacomitinib (PF-00299804), a pan-EGFR-family irreversible inhibitor is currently in several trials for NSCLC, such as phase 2trial NCT01858389 for advanced NSCLC, phase 3 NCT01360554 comparing dacomitinib and erlotinib, phase 3 NCT01774721/ARCHER 1050 comparing dacomitinib versus gefitinib, phase 3 NCT01000025 for patients with advanced NSCLC who have not responded to previous therapies, and several more. In a recent trial, treatment with dacomitinib was associated with a median PFS of 12.4 weeks compared with 8.3 weeks for erlotinib in patients with AC. However, a superior PFS with dacomitinib compared to erlotinib was not observed in other histological subtypes of NSCLC [25]. All the aforementioned EGFR inhibitors are anilinoquinazolines and engage both the mutant and wild type receptors. Thus this class of drugs share the common side effects of rash and diarrhea which arises by the binding of the EGFR receptors in the skin and gastro-intestinal tract respectively.

Other EGFR inhibitors are in various stages of development. Some of these are pyrimidines and engage only the mutant receptor. Therefore this class of drugs lack the troublesome rash and diarrhea seen with the aniliquinazolines. CO-1686 is an irreversible inhibitor of EGFR active in the presence of T790M patients, and is now in an early clinical trial involving a selected, pretreated patient population with the T790M mutation that confers resistance to first-generation inhibitors (NCT01526928). AP26113, a dual ALK/EGFR inhibitor that appears to overcome RTK-mutation-based resistance, has entered clinical testing NCT01449461. The trial recruits patients with ALK translocation and those previously treated with EGFR inhibitors that developed T790M mutation.

Inherent lack of response to EGFR inhibitors is not well understood, but presence of mutations in KRAS (codon 13) was predictive for poor response to erlotinib compared to mutant codon 12 [26]. However, KRAS and EGFR mutations are almost always mutually exclusive, and the lack of response to EGFR TKIs is most likely due to the absence of an EGFR mutation as opposed to the existence of a KRAS mutation. Some mutations in EGFR, such as insertions in exon 20 [27] and T790M (rarely found in untreated AC) confer primary resistance to EGFR.

Antibodies. Cetuximab (Erbitux), a chimeric monoclonal IgG1 antibody that blocks EGFR signaling, has been investigated in the front line in combination with platinum-based chemotherapy in advanced NSCLC through two multicenter, randomized phase 3 trials. Necitumumab and nimozutumab, other anti-EGFR antibodies, have also been investigated in several clinical trials, mostly in combination with chemotherapy regimens. A randomized phase 3 trial (SQUIRE) comparing chemotherapy (gemcitabine and cisplatin) with necitumumab versus chemotherapy alone demonstrated a significantly increased overall survival (OS) in patients with metastatic stage 4 SQCC (Eli Lilly press release, Aug 2013).

Combination Therapies. Over 100 clinical trials for recurrent or advanced NSCLC involve erlotinib in combination with other therapies. Results of a trial using combination of erlotinib and tivantinib/ARQ 197, a non-ATP-competitive inhibitor of MET, showed clinical activity in patients with NSCLC, with 6 of 8 patients achieving stable disease [28]. One study explored combination of erlotinib with HDAC inhibitor entinostat in patients with advanced NSCLC that had progressed after chemotherapy. Although the combination regimen did not demonstrate a significant survival advantage in the overall population, it showed a synergistic effect in the subset of patients with high E-cadherin expression [29]. A phase 1 study of the combination of erlotinib, cetuximab, and bevacizumab was well-tolerated and demonstrated antitumor activity in heavily pretreated patients with NSCLC [30]. A meta-analysis of results of studies of erlotinib alone versus combination targeted therapies concluded that the latter are superior over erlotinib monotherapy as second-line treatment for advanced NSCLC [31].

A phase 3 randomized trial explored addition of erlotinib to the doublet chemotherapy with cisplatin/gemcitabine as a - treatment in patients with advanced NSCLC. Addition of erlotinib significantly prolonged PFS in patients with mutant EGFR [32]. Addition of afatinib to the same regimen of chemotherapy in a randomized phase 3 trial had a strong positive effect on PFS [24].

There are a number of other trials evaluating the efficacy of combining first- or second-generation EGFR inhibitors with other molecularly targeted agents. Some of ongoing combination therapy trials with erlotinib are: NCT01294306 - erlotinib and AKT inhibitor MK2206; NCT01859026 - erlotinib and MEK inhibitor MEK162, in EGFR and KRAS mutant NSCLC; NCT00994123 – erlotinib and MM-121, an anti-ERBB3 antibody (see below, under ERBB3 section), NCT01454102 – erlotinib and nivolumab (anti-PD-1 antibody), NCT00966472 - erlotinib with Rosurastatin, NCT01186861 - erlotinib and linsitinib/OSI-906 (IGF1R inhibitor); NCT01708954 - erlotinib with Aurora A inhibitor MLN8237; NCT01471964 – erlotinib and mTOR ihnibitor CC-223. Afatinib is currently being evaluated in combination with nimozutumab (anti-EGFR antibody) in NCT01861223, with cetuximab (anti-EGFR antibody) in NCT01090011, with the HSP90 inhibitor AUY922 in NCT01259089, and with sirolimus/rapamycin (mTOR inhibitor) in NCT00993499.

As already mentioned above, anti-EGFR antibodies are also analyzed in combination trials. For example, NCT01451632 examines the effects of cetuximab with MM-121 (anti-ERBB3 antibody) and chemotherapeutic agent irinotecan. Necitumumab is also currently being investigated in six trials involving combination chemotherapy.

##### Resistance to EGFR inhibitors

As noted, EGFR inhibitors have a significantly better effect on PFS compared with conventional chemotherapy [33]. However, nearly all patients with EGFR-mutant-AC develop resistance to the first-generation inhibitors erlotinib and gefitinib, with a median PFS of 14 months [34]. Several mechanisms have been implicated in resistance to EGFR inhibitors, and they were reviewed recently [35-37].

The most common mechanism of acquired resistance to erlotinib or gefitinib is emergence of the “gatekeeper” mutation in EGFR itself, which likely indicates the “addiction” of lung cancer to EGFR signaling. A single amino acid change, T790M in exon 20 of the EGFR gene, is found in about 50% to 60% of patients developing resistance [38]. EGFR TKIs (such as erlotinib or gefitinib) are selective inhibitors of EGFR's kinase domain, that work by competing with ATP at the ATP-binding site, thereby preventing autophosphorylation and activation. The T790M mutation affects the gatekeeper residue in the catalytic-kinase domain and confers drug resistance by increasing EGFR's affinity for ATP – thus reducing the potency of the ATP-competitive kinase inhibitors [39]. Interestingly, the development of a T790M mutation may actually confer a relatively improved survival, as tumors that acquire it appear to be less aggressive than tumors with EGFR TKI resistance due to other mechanisms [40].

The other common mechanism of acquired resistance to EGFR tyrosine kinase inhibition involves amplification of RTK c-MET [41], a proto-oncogene that encodes a protein also known as hepatocyte growth factor receptor (HGFR),. Interestingly, a subpopulation of cells with amplified MET might preexist in tumors – i.e., MET amplification occurs in limited areas prior to treatment with EGFR inhibitors [42]. This might be due to increased levels of HGF that apparently accelerate development of MET amplification *in vitro* and *in vivo* [42]. Activation of MET promotes epithelial to mesenchymal transition or EMT [43].

EMT has been heavily implicated in the resistance of NSCLC to EGFR inhibitors and could involve upregulation of another RTK AXL. AXL drives a mesenchymal phenotype, and resulting tumors acquire sensitivity to AXL inhibitor SGI-7079 [44]. IGFR-1 could also promote EMT in tumors with EGFR mutations, and this involves TGFα-1 signaling rather than MET or AXL hyperactivaiton [45].

In addition to EMT, PIK3CA mutations, and conversion to small cell lung cancer histology are other mechanisms that have been implicated in resistance to EGFR inhibition [46]. Serial biopsies revealed that these genetic mechanisms of resistance were lost in the absence of the continued selective pressure of EGFR inhibitor treatment, and such cancers were sensitive to a second round of treatment with EGFR inhibitors [46].

Another mechanism of resistance is amplification of HER2 reported to occur in 12% of tumors that developed resistance to EGFR inhibitors [47]. HER2 amplification and EGFR (T790M) were mutually exclusive in this setting. Afatinib (second-generation EGFR inhibitor) and cetuximab (anti-EGFR antibody) significantly inhibit HER2 phosphorylation *in vitro*, suggesting that tumors acquiring resistance to erlotinib should be tested for HER2 status and potentially treated with a pan-EGFR TKI inhibitor such as afatinib [47].

An extensive study examined potential involvement of mutations in BRAF, NRAS, KRAS, and MEK1 in a large number of resistant tumors, and found no evidence of secondary mutations in these genes with the exception of BRAF, where mutations were identified in 2 of 195 (1%) biopsies [48]. However, reduced expression of the negative regulator of RAS proteins, NF1, was implicated in both intrinsic and acquired resistance to EGFR inhibitors that could be rescued with MEK1 inhibitor [49].

Preclinical studies with NSCLC cells selected *in vitro* for resistance to EGFR inhibitions indicated other potential mechanisms of acquired resistance, such as increased expression of FGF2 and FGFR1, in an autocrine bypass loop [50].Another study has identified an acquired amplification of the adaptor protein CRKL (that has known oncogenic properties) in an NSCLC patient that developed resistance to erlotinib [51].

Deubiquitinating enzymes that prevent ubiquitination-triggered degradation of RTKs could become a new target in forestalling resistance to RTK inhibitors. Silencing or pharmacological inhibition of USP8 deubiquitinase, relevant in particular to the stability of RTKs such as EGFR and MET, was shown to induce death of gefitinib-resistant NSCLC cells *in vitro* and *in vivo* [52].

17-DMAG (Hsp90 inhibitor) and belinostat (histone deacetylase inhibitor) alone and particularly in combination were shown to be efficacious in a setting of resistance to EGFR inhibitors conferred by mutations in EGFR or PTEN [53]. These pathways already are and will be further interrogated in clinical trials.

##### Addressing drug resistance in EGFR mutant NSCLC

Second Generation EGFR Inhibitors. The second-generation TKIs such as afatinib (BIBW2992) described above irreversibly inhibit RTKs of EGFR family, as well as the T790M variant of EGFR [21, 54]. As mentioned above, afatinib has been evaluated in the LUX-Lung trials, with improvement in PFS reported in patients with EGFR-activating mutations, as both the first- and second/third-line therapies compared to chemotherapy. But some other results indicate limited activity of the second generation of EGFR inhibitors in the setting of T790 mutation [55, 56].

The novel inhibitor CO-1686 showed promising results in NSCLC patients with the T790M EGFR mutation that were previously treated with the first-line EGFR inhibitor (erlotinib or gefitinib) (NCT01526928). Resistance to CO-1686 was observed *in vitro* and could be overcome with an inhibitor of AKT [57]. AP26113, a dual ALK/EGFR inhibitor that also appears to overcome T790M-mutation-based resistance, has entered clinical testing (NCT01449461) in patients with acquired T790M. AZD9291 is another new inhibitor of EGFR including T790M variant in clinical development (NCT01802632) and has already produced partial responses in patients that progressed on other EGFR inhibitors (15th World Conference on Lung Cancer, 2013).

Some evidence indicates that targeting other RTKs of the EGFR family in combination with EGFR inhibitors might be efficient in preventing development of resistance [58]. Clinical trials addressing this possibility are listed above, in “Combination Treatments.” In particular, targeting ERBB3 is of clinical interest due to its ability to strongly activate PI3K signaling.

MET inhibitors. Various drugs or antibodies capable of inhibiting MET (e.g., crizotinib, foretinib, ARQ 197, MetMAb) could, in principle, be combined with the first (erlotinib) or second (Dacomitinib/PF-00299804, afatinib/ BIBW2992) generation EGFR-TKIs. Concurrent inhibition of both may improve patient outcomes. Small-molecule inhibitors of MET and MetMAb/Onartuzumab are currently being tested in NSCLC (see MET section). However, the phase III trial of Onartuzumab combined with erlotinib in MET positive EGFR mutant NSCLC failed to improve PFS or OS in spite of the positive results from a phase II trial [59].

Hsp90 inhibitors. HSP90 is a molecular chaperone that is critical for tumor growth and proliferation. Many cancers have increased levels of active Hsp90, which is involved in protein folding. Client proteins of HSP90 include many signaling kinases such as RTKs and intracellular kinases essential for cancer cell survival, since lack of HSP90 triggers protein degradation. Hsp90 inhibitors may thus block multiple signaling pathways that are functioning aberrantly in cancer cells. Hsp90 inhibitors such as AUY922 and ganetespib (STA9090) are in many clinical trials for lung cancer. Both inhibitors showed good efficacy in preclinical models of NSCLC [60-62]. Ganetespib monotherapy showed clinical activity in heavily pretreated patients with advanced NSCLCs, particularly in patients with tumors harboring ALK gene rearrangement [63] (see “ALK” section). AUY922 is currently being evaluated in five trials for NSCLC. NCT01784640, for example, addresses specifically efficacy of AUY922 with pemetrexed in previously treated Stage IV patients. However, HSP90 inhibitors are expected to work best in combination with other drugs, and a randomized phase 2trial of ganetispib with docetaxel showed a significant improvement in overall survival in stage 4 patients (ASCO meeting 2013). A different type of inhibitor, OGX-427, an antisense oligonucleotide to HSP90, is currently in clinical testing in combination with chemotherapy (NCT01829113).

Combination therapies. A number of trials examining combination treatments to forestall or overcome resistance to EGFR inhibitors were described above. Ongoing trials for recurrent or advanced NSCLC will test the efficacy of various combination therapies including EGFR inhibitors, second-generation tyrosine kinase inhibitors, dual MET/VEGFR2 inhibitor, and targeted drugs to different proteins disregulated in lung cancer. For example, docetaxel with or without the FGFR1 inhibitor AZD4547 is currently being tested in both NSCLC and squamous cell carcinoma of lung (NCT01824901). NCT01121575 explores combination of PF 02341066 (Crizotinib) with the pan-HER inhibitor PF 00299804. NCT01337765 examines MEK and PI3K/mTOR inhibitor combination in patients who progressed on EGFR inhibitors. NCT01487265 will explore combination of PI3K inhibitor BKM-120 and erlotinib in patients that previously responded to erlotinib but developed resistance. An ongoing phase 1 trial explores the combination of afatinib with anti-EGFR antibody cetuximab in the setting of acquired resistance to erlotinib or gefitinib (NCT01090011).

##### EGFR inhibitors in tumors with wild-type EGFR

Identification of patients with wild-type EGFR tumors who may benefit from treatment with an EGFR inhibitor is a clinically important question. The two first-generation EGFR inhibitors differ in their selectivity towards tumors with wild-type EGFR. Erlotinib has demonstrated activity in pre-treated patients with EGFR wild-type tumors [64], with modest improvement in survival outcomes as switch-maintenance therapy [65]. In contrast, gefitinib has only demonstrated activity in patients with activating mutations in EGFR. Dacomitinib showed a superior PFS to erlotinib in patients with wild- type (and mutant) EGFR [25].

An approved test known as VeriStrat provides likely responsiveness to EGFR inhibitory therapies in the absence of EGFR mutations. VeriStrat utilizes mass spectrometry to evaluate tumor EGFR ligand levels and predict patient response and survival outcome to erlotinib and other EGFR inhibitors from serum samples [66]. Several recent studies have indicated that VeriStrat classification has significant power to predict response to EGFR inhibitors for several cancer types [67, 68]. The “VeriStrat good” patients had increased survival after erlotinib treatment compared to the “VeriStrat poor” group [69]. A later study found that VeriStrat has a prognostic role in patients with advanced, nonsquamous NSCLC treated with erlotinib and bevacizumab in the first line [70].

However, the use of RTK inhibitors in wild-type EGFR NSCLC patients remains a subject of controversy, with some investigators advocating it [71, 72], while others provide data analysis indicating no benefit in this large heterogeneous population of patients [73]. A large randomized trial has showed the superior efficacy of docetaxel over erlotinib in wild-type EGFR patients [74]. It appears that grouping together EGFR wild-type NSCLC as a category is misleading, and should be replaced by further molecular analysis of these tumors with a goal of identifying the clinically important genetic alterations. Indeed, the current (2013) ASCO guidelines state: “in unselected patients, evidence is insufficient to recommend single-agent erlotinib or gefitinib as first-line therapy… if EGFR mutation status is negative or unknown, cytotoxic chemotherapy is preferred.”

##### EML4-ALK

The EML4-ALK oncogene is a fusion between echinoderm microtubule-associated protein-like 4 (EML4) and anaplastic lymphoma kinase (ALK) [75]. The fusion generates an overexpressed and activated tyrosine kinase, whereas normal lung tissue does not express ALK. EML4-ALK fusions are found in about 3% to13% of NSCLC patients [76], and are largely mutually exclusive with alterations in other RTKs or KRAS based on analysis of almost 1700 tumors [77]. Patients with EML4-ALK mutant tumors are characteristically younger, and like patients with EGFR-mutated NSCLC, they typically are never smokers with AC histology [78].

EML4-ALK oncogene does not involve either PI3K or MAPK pathways in its transforming activity, which suggests lack of utility of inhibitors of these pathways in treating patients with the ALK fusions. However, inhibition of molecular chaperone HSP90 leads to degradation of EML4-ALK and a rapid, but transient, regression of tumor growth in a mouse model [79].

##### Potential therapeutic approaches for EML4-ALK

ALK inhibitors. Crizotinib (Xalkori) was approved by the FDA for ALK-positive NSCLC in 2011. A large phase 3 trial has demonstrated the superiority to standard chemotherapy in patients with previously treated advanced NSCLC with ALK rearrangement [80]. A new inhibitor, LDK378, is in development, having received the FDA Breakthrough Therapy designation in March 2013. This program, introduced in the summer of 2012, is intended to expedite the development and review of drugs that treat serious and life-threatening conditions and that have demonstrated substantial improvement over existing therapies on at least one clinically significant endpoint. This designation for LDK378 was based on phase 1 trial results data that showed marked responses in a majority of patients with ALK-positive NSCLC. An overall response rate of 80% was observed in the patients whose tumor progressed on crizotinib treatment. LDK378 is currently being tested in a number of clinical trials, including NCT01685060, NCT01685138, NCT01283516, NCT01634763, NCT01828112, NCT01828099, and expanded treatment protocol trial NCT01947608 – both for patients previously treated with crizotinib and for treatment-naïve patients. Four more trials will start recruiting patients soon. There are several other ALK inhibitors currently evaluated in clinical trials, including X-396 that is being tested in the phase 1 trial NCT01625234. RO5424802/Alectinib is tested in phase 1/2 trial (NCT01588028) for ALK rearranged tumors, and in a phase 2 trial NCT01801111 for patients who failed on crizotinib. Preliminary results showing excellent activity were reported from a clinical trial in Japan [81], and RO5424802 received the Breakthrough Therapy designation in October 2013. AP26113, an ALK inhibitor from Ariad, was reported to show efficacy based on preliminary results of trial NCT01449461. The drug was effective in patients previously treated with crizotinib, in patients with brain metastases and, interestingly, in EGFR-mutant patients including T790M.

##### HSP90 Inhibitors

The Hsp90 inhibitor ganetespib, in a preclinical study conducted *in vitro* and in animals, induced loss of EML4-ALK expression and depletion of multiple oncogenic signaling proteins in ALK-driven NSCLC cells [82]. Ganetispib had a greater *in vitro* potency and better antitumor efficacy compared to crizotinib [82]. Ganetespib (STA-9090) is being tested in clinical trials for ALK-positive lung cancer patients, alone (NCT01562015) or in combination with crizotinib (NCT01579994). A completed phase 2 trial of ganetespib showed clinical activity in heavily pretreated patients with advanced NSCLCs, particularly in patients with tumors positive for EML4-ALK [63]. In preclinical models, Ganetispib was shown to be not only effective in ALK positive adenocarcinoma, but was able to overcome resistance to crizotinib [82]. Another HSP90 inhibitor, AUY-922 is also in clinical trial for advanced ALK-positive patients (NCT01752400). NCT01772797 examines the safety of LDK378 in combination with AUY-922. Phase I/II clinical trial NCT01712217 will explore HSP90 inhibitor AT13387 alone or in combination with crizotinib.

##### Resistance to crizotinib

Patients treated with crizotinib usually develop resistance to it, and the mechanisms of this appear to be heterogeneous. Several patterns were described: secondary (gatekeeper) mutations in ALK that render the kinase resistant to crizotinib; increased ALK copy number, EGFR L858R mutation and high levels of amphiregulin in sera of resistant patients [83]. Another study of resistance to crizotinib also identified secondary mutations in ALK, in EGFR, and in KRAS [84]. Although crizotinib was ineffectual against EML4-ALK harboring the gatekeeper mutation, two structurally different ALK inhibitors, NVP-TAE684 and AP26113, were highly active against the resistant cancer cells *in vitro* and *in vivo* [85]. As mentioned above, RO5424802 is in clinical trial for patients who have failed crizotinib. A new ALK inhibitor in preclinical development, PF-06463922, showed activity in crizotinib-resistant tumors in mouse models, and was able to cross blood-brain barrier. It is about to enter clinical testing (NCT01970865).

##### MET

MET (mesenchymal-epithelial transition factor) RTK, also known as hepatocyte growth factor receptor HGFR, is a proto-oncogene with important implications in NSCLC [86]. MET activation induces specific phosphorylation of several tyrosine residues, which, in turn, activates multiple downstream signaling pathways, including RAS/MAPK, PI3K, and SRC kinase pathways (reviewed in [87]). MET promotes epithelial-mesenchymal transition (EMT), at least in part through activation of SRC. Elevated levels of HGF and intratumoral MET expression have been associated with a more aggressive biology and a worse prognosis in NSCLC. Disregulation of MET pathway leads to cell proliferation, cell survival, angiogenesis, invasion, and metastasis.

MET disregulation can occur through a variety of mechanisms including c-MET overexpression, activation, gene amplification, and overexpression of the c-MET ligand hepatocyte growth factor (HGF). Tests for the detection of these abnormalities are not FDA approved, nor is c-MET considered a predictive marker that informs clinical decision making.

MET mutations have been observed in about 5% of treatment-naive NSCLC [88], mainly within exons 2 and 14, outside of the kinase domain. Mutations in MET are observed more often in smokers [89]. The MET mutation N375S was detected as a germline mutation in a high proportion of East Asian samples, and was correlated to incidence of squamous-cell carcinoma [89].

MET amplification, as mentioned above, is one of the mechanisms of the acquired resistance to EGFR inhibitors and occurs in about 20% of treated patients [90], but only in 1.4% of treatment-naïve patients [91], exclusively in AC. A splice mutation in MET deleting the juxtamembrane domain for binding the c-Cbl E3-ligase was observed in 3.3% of patients [91]; normally such binding leads to ubiquitination and receptor degradation, and loss of this domain leads to MET activation.

MET overexpression has been observed in 25% to 61% of NSCLC patients [92, 93]. The MET ligand HGF is also expressed by tumor cells, with moderate overexpression observed in a high proportion of lung tumors [93, 94].

##### Potential therapeutic approaches for MET

MET inhibitors. Several targeted therapies for the treatment of aberrant MET activity are currently in clinical testing. MET inhibitor tivantinib (ARQ 197), in combination with erlotinib in previously treated patients, has shown better activity versus erlotinib alone in phase 2 study [95]. Response rate was higher in KRAS-mutant NSCLC patients [95]. A randomized phase 3 study, NCT01395758, was completed, and did not meet its primary endpoint. However, further subset analysis is currently being carried out. Tivantinib might have an off-target activity.

Multi-RTK inhibitor Cabozantinib (XL184) is in active or planned trials for NSCLC, as a single therapy or with erlotinib (phase 2 trials NCT01866410 and NCT01708954 for previously treated NSCLC). Foretinib, a dual MET/VEGFR inhibitor, was in a dose escalation study in various solid tumors, but the best responses observed were stable disease [96]. Foretinib is tested alone or in combination with erlotinib in NCT01068587. Specific MET inhibitors EMD 1214063 and EMD 1204831 are in safety testing in various solid tumors. Nonspecific inhibitor of MET MGCD265 is being tested in combination with either erlotinib or docetaxel in a phase 1/2 trial NCT00975767 for NSCLC.

MET antibodies. A single-arm antibody directed against the extracellular domain of MET, called MetMAb (onartuzumab), has been tested in a randomized phase 2 trial comparing its combination with erlotinib versus erlotinib alone as a second- or third-line treatment. Patients whose tumors had high c-MET expression had significantly longer PFS with MetMAb plus erlotinib compared with erlotinib monotherapy, and significantly longer OS (12.6 versus 4.6 months). In the cohort of patients who received MetMAb, median OS was much better for patients with high c-MET expression than for those negative for c-MET (12.6 versus 5.5 months) [97]. Several clinical trials are evaluating MetMAb in different settings of NSCLC, including a prospective, randomized phase 3 study designed to validate the MetMAb findings from the phase 2 study (NCT01456325), and a study in SQCC comparing chemotherapy combination alone to chemotherapy and MetMAb (NCT01519804).

*HGF antibodies*. Two anti-HGF antibodies were tested in lung cancer patients. AMG-102 (rilotumumab) is a human monoclonal antibody that showed some activity in an early trial in patients with advanced solid tumors [98]. AMG-102 is currently being tested in combination with erlotinib (NCT01233687). A second HGF antibody AV-299 was tested in phase 1 trial without promising results. Based on this initial experience, there is currently limited interest in clinical development of anti-HGF drugs.

##### ERBB2/HER2

HER2 is a member of the EGFR/ERBB family of RTKs, best known for its role in breast cancer, where it is targeted by the antibody trastuzumab. HER2 is unique in the EGFR family because it does not bind any known ligands of the EGF family, but exists in an unliganded, active confirmation. Its function is exerted via heterodimerization with other family members. Mutations in HER2 were identified in 2% to 4% of NSCLC examined [99-101]. Most mutations occur in exon 20 affecting the ATP-binding pocket. A recent study has confirmed the previously reported higher rate of association of HER2 mutations with lung cancer in women and nonsmokers [102]. Tumors with the HER2 mutation were mostly adenocarcinomas, with the-HER2 mutation almost always an exclusive driver [102]. Even though mutations are rare, HER2 more frequently shows a moderately increased copy number in AC, with different frequencies reported in different studies [103-105], and around 10% in the large NGS study [2].

Mutations aside, ERBB2 was implicated in resistance of NSCLC cell lines to EGFR-directed antibody cetuximab *in vitro*. Activation of ERBB2 signaling in cell lines through ERBB2 amplification or through heregulin up-regulation resulted in persistent activation of extracellular signal-regulated kinase (ERK1/2) and cetuximab resistance [106].

##### Potential therapeutic approach for HER2

Thus far there has been limited clinical experience with HER2 inhibitors in treatment of NSCLC, but a number of trials are ongoing. Responses were observed with trastuzumab and afatinib [102, 107] but not with other EGFR-family-targeted drugs such as gefitinib or lapatinib [108, 109]. Neratinib is a second-generation, irreversible inhibitor of HER2 that is currently being tested in NSCLC (NCT01827267). A dose-finding study of neratinib combined with temsirolimus produced some clinical responses in HER2 mutant NSCLC patients [110]. MGHA22 is an anti-HER2 antibody with an improved characteristic in the Fc domain that enables enhanced binding of to the Fc-gamma receptor on NK and macrophages for antibody-directed cell lysis. MGHA22 is currently being trialed in NCT01148849 and NCT01195935. Trial NCT01526473 is testing a new vaccine, HER2 VRP or AVX901, based on Venezuelan equine encephalitis virus. An attenuated strain of VEEV, in which 3 of the 7 viral genes were substituted with a truncated HER2 gene to create a self-amplifying replicon RNA, will be used to vaccinate patients with HER2-expressing cancers.

##### ERBB3

This RTK of the EGFR family might be involved in 2% of SQCC [4], though there is no direct evidence of functional significance of these mutations yet. ERBB3 is unusual in that it lacks a kinase domain of its own, but it heterodimerizes with other members of the EGFR family, leading to potent activation of the PI3K pathway (Schoeberl et al., 2009) and providing a strong rationale for the inhibition of this RTK (reviewed in [111]). The rationale for clinical development of anti-ERBB3 drugs in combination with other EGFR family inhibitors is based on the ability of EGFR family members to heterodimerize with ERBB3, leading to activation of ligand-dependent signaling [58]. Dual inhibition of ERBB3 and EGFR shows synergistic effects *in vitro* in cell lines derived from pelural effusion of lung adenocarcinoma patients [112]. Interestingly, while ERBB3 supports PI3K signaling in epithelial cells, *in vitro* this leads to downregulation of Erbb3 and rewiring of PI3K activation [113].

##### Potential therapeutic approach for HER3

Clinical studies of anti-ERBB3 antibody MM-121 are ongoing in NCT00994123 and NCT01451632, including combination study with erlotinib. NCT01451632 examines effects of MM-121 with cetuximab (anti-EGFR antibody) and chemotherapeutic agent irinotecan.

##### ROS1

ROS1 is a transmembrane proto-oncogene RTK of the insulin-receptor family that is aberrantly expressed in neoplasms of the central nervous system. ROS-1 translocations are observed in glioblastoma and other tumors and, infrequently, in NSCLC, in about 1% to 2% of patients (reviewed in [114]). Patients with ROS1-mutant tumors are characteristically young nonsmokers. The fusions of ROS with SLC34A2 and CD74 were identified in a phosphoproteomic screen of tyrosine kinase activity in lung cancer [115], and have been observed predominantly in AC [116, 117]. ROS1 rearrangement defines a molecular subset of NSCLC with clinical characteristics that are similar to those observed in patients with ALK-rearranged or EGFR-mutated NSCLC [117]. They are mutually exclusive with EGFR, KRAS, and EML4-ALK mutations.

##### Potential therapeutic approach for ROS1-positive NSCLC

ROS1-positive NSCLC represents a unique subset of patients for whom the c-MET/ALK inhibitor crizotinib has shown potential as a very effective therapeutic strategy. A phase 1 trial evaluating crizotinib in advanced solid tumors that possess the ROS1 translocation is currently ongoing (NCT00585195). The investigational ALK inhibitor LDK378, fast-tracked by FDA, might also have an inhibitory effect on ROS1, though it is not presently investigated specifically in ROS1-positive patients. AP26113 is a dual EGFR/ALK inhibitor in clinical development (NCT01449461), and the ongoing trial will recruit patients with ROS1-positive NSCLC as well. ALK/ROS1 inhibitor PF-06463922 is in early phase trial NCT01970865. An efficacy trial of HSP inhibitor AUY922 is recruiting patients with various RTK alterations including ROS1 fusion. Trial NCT01712217, which explores crizotinib in combination with HSP90 inhibitor AT13387, is recruiting patients with both ALK- and ROS1-positive NSCLC who have failed crizotinib. A new ALK/ROS inhibitor PF-06463922 showed activity in crizotinib-resistant tumors in mouse models and is about to enter clinical testing (NCT01970865). Resistance to crizotinib in a patient with CD74-ROS1 fusion was already reported, apparently as a consequence of a *de novo* mutation of G2032R in the ROS1-kinase domain [118]. Preclinical studies showed that ROS1-positive cancer cells, including the G2032R variant, are sensitive to MET/VEGFR2 inhibitor foretinib, strongly suggesting that foretinib could be the drug of choice for ROS1-positive NSCLC [119].

##### RET

RET (rearranged during transfection) fusions are considered to be driver mutations in lung cancer because they are not found in association with changes in EGFR, KRAS, ALK or HER2. RET rearrangements are common (30%) in papillary thyroid carcinomas [120], and mutations are found in 50% of medullary thyroid carcinomas [121]. RET is an RTK involved in neural crest development. RET ligands belong to the glial cell-derived neurotrophic (GDNF) family, and upon engagement, RET induces activation of multiple signaling pathways: PI3K, MAPK, JNK, p38, SRC, ERK5, PLC-γ, and STAT [122]. The most common fusion partner of RET in NSCLC is KIF5B, a gene in the family of motor proteins, kinesins [117, 123, 124]. Other partners have been identified: CCDC6 [117] and NCOA4 [125]. Translocations are observed in a small percentage of patients (1% to 2%), but stratification by smoking and age results in a higher percentage of RET translocations. Patients with RET translocations tend to be younger nonsmokers and have poorly differentiated tumors [125].

##### Potential therapeutic approach for RET-positive adenocarcinoma

The multikinase inhibitor vandetanib (ZD6474) is an inhibitor of RET and VEGFR1, and is approved for medullary thyroid cancer with RET fusions. Its activity in lung adenocarcinoma with translocated RET is currently undefined. None of the trials conducted thus far included sequencing to determine the status of RET, but one ongoing trial of vandetanib, NCT01823068 (in South Korea), will recruit patients whose tumors harbor a RET translocation. Vandetanib is also being tested in combination with the MEK inhibitor selumetinib NCT01586624. Significant increases in PFS were reported in three out of three patients with RET-rearranged AC in a small trial of cabozantinib [126]. Other multikinase RTK inhibitors are also being tested in RET-positive AC, including cabozantinib (XL-184) in NCT01639508, ponatinib in NCT01813734, and sunitinib in NCT01829217.

##### FGFR1

FGFR (fibroblast growth factor receptor) pathway signaling normally contributes to the physiologic processes of tissue repair, hematopoiesis, angiogenesis, and embryonic development. Focal amplification of FGFR1 was detected in 21% to 22% of SQCC tumors according to two reports [127, 128], but is quite rare in AC (1% to 3%) [2, 128]. Other members of the family (FGFR2 and FGFR3) might be also activated in SQCC at low frequencies of 2% to 3% [4]; mutations in these receptors are oncogenic *in vitro* and *in vivo*, and are sensitive to RTK inhibitor pazopanib [129]. FGFR1 is the only RTK amplified at appreciable frequency (6%) in SCLC [5].

SQCC tumors with amplified FGFR1 were found to have high expression of MYC in 40% of cases, and this subset is more sensitive to inhibition of FGFR, providing a potential response-predictive biomarker [130].

##### Potential therapeutic options to FGFR alterations

FGFR inhibitors PD173074 and AZD4547 inhibit proliferation of SQCC *in vitro* and tumor growth *in vivo* [128, 131]. AZD4547 is under clinical investigation in SQCC, including a phase 2 trial NCT01795768 in molecularly selected patients, a phase 1 trial NCT00979134, and a phase 1/2 trial with docetaxel for recurrent NSCLC and SQCC (NCT01824901). BGJ 398 is a pan-FGFR inhibitor being investigated in a dose-escalation (safety) trial (NCT01004224) for FGFR-amplified malignancies. The multi-RTK and SRC-family inhibitor BIBF 1120 (vargatef) is currently being explored in Korean patients with small cell lung cancer (SCLC) in NCT01441297. Dovitinib (TKI258) is an inhibitor of FGFR3 and potentially other FGFRs, which is currently being tested in patients with advanced NSCLC or colorectal cancer (NCT01676714) and in patients with SQCC (NCT01861197) and various tumors phase 2 NCT01831726). Nintedanib is entering phase 2 studies in patients with FGFR1 amplified NSCLC (NCT01948141). BAY1163877, a pan-FGFR inhibitor, is entering a safety study in patients with FGFR-amplified cancers (NCT01976741). GSK3052230, an antagonist of FGFR receptors, is a fusion protein composed of the extracellular domain of FGFR1 and the Fc portion of IgG1. It is currently in a phase 1 study NCT01868022 as a monotherapy or in combination with chemotherapy in SQCC patients with alterations in FGF receptors.

##### DDR2

Discoidin domain receptors (DDRs) are RTKs that belong to the same family as EGFR. However, DDRs are unique because they have native collagens as their ligands. DDR2 mutations, identified by Sanger sequencing as the tyrosine kinome in 11 of 290 (3.8%) SQCC samples, may alter kinase activity, ligand binding, or DDR2 localization [132]. A squamous cell lung cancer patient with a response to dasatinib and erlotinib treatment harbored a DDR2 kinase domain mutation [132]. A phase 2 trial NCT01514864 is currently examining dasatinib in NSCLC with DDR2 mutation. However, previous studies of dasatinib in unselected patient populations showed significant toxicity and lack of benefit [133].

##### IGF1R

Insulin growth factor 1 receptor is an abundantly expressed RTK, frequently elevated in lung cancer, particularly in SCLC and certain NSCLC patients. There has been interest in developing IGF1R-targeting clinical options, but the validation of the efficacy of IGF1R-targeted agents in large clinical trials had largely failed. Considering the scarcity of targeted agents for SCLC, IGF1R is certainly an attractive target. IGF1R activates PI3K and MAPK signaling, and recently it was shown that IGF1R is involved in signaling from mutant KRAS, which also makes it a very attractive target in KRAS-mutated NSCLC. NSCLC lines with activated KRAS depend on the basal IGF1R activity for activation of PI3K pathway [134]. Inhibitors of IGF1R are active in cell lines with mutant, but not wild type, KRAS. Moreover, inhibition of both IGF1R and MEK *in vitro* and *in vivo* in NSCLC with mutant KRAS is highly cytotoxic [134].

##### Potential therapeutic options for activation of IGFR1

Anti-IGF1R antibody figitumumab (CP-751,871) in combination with carboplatinum-paclitaxel was tested in NSCLC with some initial promising results [135], but subsequent trials were discontinued because of futility and unacceptable toxicity. Another antibody, cixutumumab, was in clinical testing combined with chemotherapy, but its development was discontinued based on negative results of a randomized phase 2 trial. The monoclonal antibody AMG-479 (ganitumab) was tested in several clinical trials, which were either terminated or did not meet endpoints, with only one trial, NCT01327612, still ongoing. The combination of erlotinib with the IGF1R monoclonal antibody, R1507, did not reveal a statistically significant improvement in PFS or OS in NSCLC [136]. AXL1717, or picropodophyllin, is a small-molecule inhibitor of IGF1R, which is currently tested in patients with SQCC in trial NCT01561456. OSI-906/linsitinib is another small-molecule. dual-kinase inhibitor of both IGF1R and the insulin receptor. OSI-906 is in randomized trials for SCLC (NCT01533181, NCT01387386) and NSCLC (NCT01221077, NCT01186861).

##### NTRK1

About 3% of NSCLC tumors that have no known driver mutation were found to harbor oncogenic fusions of nerve growth factor receptor NTRK1 with MPRIP or CD74 [137]. Translocations were found in women never-smokers. NTRK1 encodes protein TRKA with tyrosine kinase activity. Lung cancer cells with NTRK1 fusions were sensitive to kinase inhibitors of TRKA, and one of them showed modest activity in a patient with NRFK1 translocation [137]. Crizotinib has been shown to have modest activity against tumors that have NTRK1 fusion.

#### 1.2 RAS PATHWAY

##### KRAS

KRAS belongs to a family of small GTPases (proteins with intrinsic GTPase activity) that regulate cellular behavior in response to extracellular stimuli. Ras-regulated signal pathways control processes such as actin cytoskeletal integrity, proliferation, differentiation, cell adhesion, apoptosis, and cell migration via the MAPK and PI3K pathways. KRAS mutations are observed in close to 30% of adenocarcinomas of the lung, but are uncommon in SQCC (about 5%) [138]. KRAS mutations are found in majority of mucinous adenocarcinomas [139, 140]. Point mutations at codons 12, 13, or 61 in the KRAS oncogene lead to constitutive activation of KRAS protein via changes at the GTP binding domain, which prevents the conversion of GTP to GDP. KRAS mutations are seen more frequently in smokers (26%) versus never-smokers (6%) [141]. In clinical trials, the prognostic significance of KRAS mutations in NSCLC has been variable, with some trials and pooled analyses reporting worse overall prognosis and others failing to identify any difference in survival outcomes [142, 143]. The presence of a KRAS mutation has not consistently resulted in different survival outcomes [141, 144].

Despite widespread KRAS mutations in a variety of cancers, this protein has not been successfully targeted therapeutically; however, several approaches are being tested in clinical trials. KRAS-mutant tumors might be uniquely sensitive to combinations of IGF1R and MEK inhibitors [134]. KRAS-driven lung tumors are dependent on the activity of the PI3K pathway, as shown recently in a mouse model. KRAS interacts with the p110a subunit of PI3 kinase, and disruption of this association impedes both development and growth of already established tumors – in particular, in combination with MEK inhibition [145]. Another preclinical study based on shRNA screening identified BCL-XL and MEK inhibition as a potentially successful strategy for treatment of KRAS-mutant adenocarcinoma [146]. There is potential in targeting the membrane localization of KRAS, which is essential for its activity. The correct localization of prenylated KRAS depends on its interaction with prenyl-binding protein PDEα. A compound was identified in a large screen and further optimized to disrupt the interaction of PDEα with KRAS. This compound, deltarasin, inhibited growth of KRAS-mutant pancreatic cells *in vitro* and *in vivo*, and might be promising for the future drug discovery efforts [147].

Another cellular module activated by KRAS is IKK-related kinases TBK and IKKα, which promote KRAS driven tumorigenesis by upregulating production of cytokines CCL5 and IL6. Compound CYT387 previously known as inhibitor of JAK, is also a potent inhibitor of TBK/IKKα with anti-tumor activity in mouse models [148].

Lastly, the “untargetability” of KRAS has been challenged recently, with development of specific small-molecule inhibitors against KRAS (G12C) that have undergone preclinical testing, and hopefully will come to clinical fruition [149].

##### Potential therapeutic approach for KRAS mutated NSCLC

**MEK and mTOR inhibitors** Preliminary evidence suggests that MEK inhibition may be an effective strategy in treating KRAS-mutated tumors and a number of clinical trials are currently studying this approach. The MEK inhibitor selumetinib showed promising results in KRAS-mutated NSCLC when combined with docetaxel in a phase 2 clinical trial [150]. The combination treatment was associated with improved PFS and a trend to longer OS. Another MEK inhibitor, trametinib, is currently trialed in combination with several chemotherapeutic drugs or erlotinib (NCT01192165). A recent study identified the basis of different activity of MEK inhibitors in BRAF versus KRAS mutant cancers. Unlike trametinib-like inhibitors, which inhibit phosphorylated MEK and are effective in the setting of BRAFV600 mutants, the new class of inhibitors, like GDC-0623, inhibit feedback activation of MEK by RAF, and are therefore more efficacious in the setting of mutant KRAS [151]. GDC-0623 is currently in a phase 1 clinical trial NCT01106599.

The combination of erlotinib and tivantinib (MET inhibitor) in NSCLC showed clinical activity in patients with KRAS-mutated cancers, even though the endpoints of the trial were not achieved [95]. Tivantinib with erlotinib is currently being tested versus chemotherapy in NCT01395758. Lastly, the combination of inhibitors targeting both MAPK and AKT/PI3K pathways are currently being tested in early-phase trials NCT01363232 (MEK162 and BKM120) and NCT01449058 (MEK162 and BYL719).

**HSP90 inhibitors**. Another approach to targeting KRAS-mutated tumors clinically involves inhibition of the molecular chaperone Hsp90. Retaspimycin HCL is currently being tested with docetaxel (NCT01362400), and everolimus (mTOR inhibitor) in NCT01427946; however, preliminary reports on the docetaxel combination are disappointing. The already-mentioned inhibitor AUY922 is currently being examined in a single-arm study in NSCLS patients with KRAS, EGFR mutations, and EML-ALK translocation (NCT01124864).

REOLYSIN is a proprietary formulation of human reovirus, a naturally occurring oncolytic virus that preferentially replicates in cancer cells with activated KRAS. Reolysin in combination with carboplatinum and paclitaxel has been evaluated in several solid tumors. REOLYSIN and chemotherapy are currently in several phase 2 clinical trials including NSCLC with mutant KRAS or activated EGFR (NCT00861627), SQCC (NCT00998192), and as salvage therapy in previously treated NSCLC (NCT01708993). Preliminary positive results were reported by the manufacturer in September 2013, with an overall response rate of 92% in a trial involving 25 patients with SQCC.

The proteasome inhibitor bortezomib is being tested in KRAS-mutant NSCLC phase 2 trial NCT01833143.

##### NF1/neurofibromin

NF1 is negative regulator of RAS pathway activation, and a tumor suppressor. Germline mutations in NF1 deregulate both PI3K and MAPK pathways and result in familial neurofibromatosis. Mutations of NF1 in AC were discovered through sequencing of a larger number (623) of “suspected” cancer-related genes in a large number (188) of tumors [88]. NF1 is mutated in more than 10% of AC, but is also disrupted in 11% of SQCC [4]. The functional significance of NF1 in lung cancer has not been established, but because of its well-known role in many other cancers it is reasonable to assume that it's a driver event in lung cancer as well. There are no specific treatment options for NF1- disrupted tumors, but in recent studies of NF1 in melanoma, it was successfully targeted in mouse models with a combination of MEK and PI3K/mTOR inhibitors [152]. A trial of MEK inhibitor MEK162 NCT01885195 is recruiting patients whose tumors have alterations in components of the RAS-RAF-MEK pathway, including NF1.

##### NRAS, HRAS

Mutations in these well known oncogenic members of RAS family are relatively rare in AC and SQCC (<1%), except HRAS in SQCC (3%; [4]). In a very large cohort of lung tumors, NRAS mutations were present in 0.7% and most NRAS-mutated tumors were AC; these had no other driver mutations implicating NRAS in driving tumorigenesis [153]. Five of six cell lines established from NRAS-mutant AC were sensitive to MEK inhibitors selumetinib and trametinib, but not to other inhibitors tested [153].

##### RASA1

RASA1 (p120Ras GAP) is a well-known GTPase-activating protein (GAP) that functions as a negative regulator of Ras signaling downstream of several growth-factor receptors [154]. RASA1 mutations have been reported to be associated with hereditary capillary malformations, but their potential involvement in cancer only came to light through NGS-based analysis of SQCC, where its recurrent mutations were found in 4% of tumors [4]. The functional significance of this remains unknown.

#### 1.3 BRAF/MAPK PATWHAY

##### BRAF

BRAF is a serine-threonine kinase whose only known substrates are MEK1 and MEK2 in the MAPK pathway. Mutated, activated BRAF occurs in about 7% of all human cancers, but about 50% of melanomas contain BRAF mutation at V600. NSCLC tumors harbor BRAF mutations infrequently, including D594G and L596R mutation in the kinase domain [155], and G465V or G468A mutations in the G-loop of the activation domain [156]. Only about half of BRAF mutations in NSCLC are V600E [157]. The frequency of BRAF mutations in AC is ~6%, and in SQCC ~4%. BRAF mutations occur much more commonly in smokers than in nonsmokers [156].

Similarly to melanoma, BRAF mutations on their own induce a senescent phenotype, which manifests as benign adenomas in a mouse model of lung carcinogenesis. Single-copy loss of LKB1 (see below) is sufficient to drive the malignant progression of these tumors to carcinomas [158]. BRAFV600E-driven lung tumors in a mouse model depend on autophagy to sustain their glutamine metabolism and growth, indicating that targeting autophagy in this context could be clinically relevant [159].

##### Potential therapeutic approach for BRAF-mutated NSCLC

The BRAF inhibitor vemurafenib, approved for melanoma, is of limited use in NSCLC with BRAF mutations other than V600E, but might be of value in tumors with V600E mutations. Other BRAF mutations could be potentially targeted with MEK inhibitors. The RAF inhibitor sorafenib was tested in an unselected population with NSCLC, and had little if any effect. However, some clinical trials are addressing the efficacy of targeted drugs in biomarker-selected patients. Trial NCT01336634 examines BRAF inhibitor dabrafenib/GSK2118436 in BRAF-mutant NSCLC, and preliminary results suggest a promising clinical activity in pretreated patients with stage IV NSCLC carrying BRAFV600E mutation (overall response rate of 60%, BRF113928). MEK inhibitor selumetinib/AZD6244 is tested in various-BRAF mutant malignancies including NSCLC in NCT00888134. BRAF mutant tumors are also targeted using the combination of inhibitors of both MAPK and AKT/PI3K pathways in early-phase trials NCT01363232 (MEK162 and BKM120) and NCT01449058 (MEK162 and BYL719).

Resistance to BRAFV600E inhibition in NSCLC involves some of the mechanisms previously encountered in BRAF mutant melanoma. One identified mechanism involves expression of an aberrant form of BRAFV600E resistant to inhibition, and the other relies on the autocrine EGFR signaling driven by c-Jun–mediated EGFR ligand expression and leading to activation of AKT [160].

#### 1.4 PI3K PATHWAY

The phosphatidylinositol 3-kinase (PI3K)/Akt pathway is activated in many different cancers, including NSCLC. PIK3CA is the class IA PI3Kα catalytic subunit p110α. Upon growth factor stimulation, PIK3CA is activated, increasing PIP3 levels which drives phosphorylation of AKT and downstream processes such as higher protein translation (through mTOR activation), cell division, and induction of anti-apoptotic response. Activating mutations in PIK3CA have been implicated in various cancers, and occur predominantly in the helical or kinase domains of the alpha p110 catalytic subunit. PI3K pathways activity is downregulated by the tumor suppressor PTEN, a phosphatase subject to deletions or mutations in a wide variety of tumors.

##### PIK3 kinase

All available data show that direct alterations in this pathway are much more frequent in SQCC versus AC, with mutations in three genes – PIK3CA, PTEN, and AKT significantly enriched in SQCC (Table 2). In addition, PIK3CA gene amplification might occur in SQCC at a much higher frequency than do activating mutations. According to one study, PIK3CA copy-number gain was found in ~33% of squamous cell tumors and in ~6.2% of adenocarcinomas [161]. Large-scale NGS analysis of SQCC found PIK3CA mutations in 16% of SQCC [4] versus previously reported 3.6% of SQCC and 2.6% of AC [161], with exon 9 as the most frequently mutated site. PIK3CA mutations are not mutually exclusive with EGFR or KRAS mutations, but are mutually exclusive with FGFR amplification in SQCC [4].

Increase in PI3Kβ expression and loss of PTEN is also observed more often in SQCC compared to AC, suggesting that targeting of PI3K is a justified strategy in treatment of SQCC [162].

##### PTEN

Inactivating mutations or deletions of PTEN, a lipid phosphatase that dephosphorylates PIP3 and negatively regulates AKT, are observed in a wide variety of cancers, including lung cancer. PTEN mutations (in exons 5–8) were observed in 4.5% of NSCLC, significantly more frequently in SQCC (10% [163] and 8% [4]). Copy-number losses of PTEN are not often associated with NSCLC. However, loss of PTEN protein is observed much more frequently, through loss of copies or methylation – in particular, in SQCC [164]. A recent study has found that a low level of PTEN expression does not necessarily signify high activity of AKT, and that areas with different levels of AKT activation co-exist within the same adenocarcinoma of lung [165]. Apparently, signaling from IGF1R and other factors influence activity of AKT in PTEN-low cells.

##### AKT

Mutations of AKT1, a central kinase in the PI3K pathway, are rare in NSCLC, occurring in ~1.9% of cases, but are more frequent in SQCC (5.5%)[166]. The large-scale NGS analysis of SQCC identified even more frequent activating mutations in AKT3 – in 16% of tumors [4] – further underlining the significance of the PI3K/AKT pathway in pathogenesis of SQCC.

##### Potential therapeutic approaches for alterations in PI3K-AKY pathway

There are at least three potential targets for therapeutic intervention in this pathway: AKT, PI3K, and mTOR. Several drugs are in clinical development against all three targets, including mTOR inhibitors that are approved for other cancer types.

**PI3K inhibitors**. Several phase 1 and 2 trials are currently ongoing, targeting PI3K in NSCLC either alone or in combination with standard, cytotoxic chemotherapy. These agents include PX-866, a PI3K inhibitor tested with docetaxel in a phase 1/2 trial with a placebo arm (NCT01204099). A phase 1 trial of the PI3K inhibitor BKM120 in combination with chemotherapy is planned (NCT01723800), and a phase 2 single-agent trial is ongoing (NCT01297491). Trial NCT01487265 will explore the combination of BKM-120 and erlotinib in patients that previously responded to erlotinib but relapsed. PI3K inhibitor GDC-0941 is also in phase 2 combination trials (NCT01493843, NCT00974584). NCT00975182 is a phase 1 trial exploring the combination of GDC-0941 with erlotinib. Another PI3K inhibitor, SAR245408, has shown promising clinical activity in NSCLC [167].

**AKT inhibitors**. Phase 1 and 2 trials are currently recruiting that offer MK2206 – an oral, potent, allosteric inhibitor of AKT – in combination with either erlotinib or gefitinib in advanced NSCLC. NCT01294306 is a phase 2 trial that examines combination of erlotinib and MK2206; NCT01147211 explores gefitinib with MK2206. NCI is sponsoring trial NCT01306045 involving molecular profiling followed by administration of relevant targeted therapies, among them MK2206 for mutations in PI3K, AKT, or PTEN.

**mTOR inhibitors**. Multiple phase 1 and 2 trials are recruiting patients for treatment of advanced NSCLC with mTOR inhibition either alone or in combination with chemotherapy or radiation therapy. These include agents such as everolimus (>30 trials), sirolimus (>30 trials), and temsirolimus (almost 30 trials).

#### 1.5 LKB1 (STK11)/AMPK PATHWAY

##### LKB1

LKB1 is a known tumor suppressor whose germline mutations are responsible for the familial cancer syndrome known as Peutz-Jeghers, characterized by increased risk for epithelial cancers including lung carcinomas. LKB1 is an AMPK-activating kinase, and as such controls AMPK function as energy sensor and master regulator of cellular growth and metabolism. AMPK regulates activity of mTORC by phosphorylating TSC1 in one of the AMPK-governed pathways, which also include regulation of OXPHOS and autophagy.

LKB1 mutations are found in 15% to 30% of NSCLC, and half of these tumors have concurrent mutations in KRAS [168]. Another study found inactivation of LKB1 in 34% and 19% of 144 human lungs in AC and SQCC, respectively [169]. Inactivation of LKB1 on the background of oncogenic KRASG12D in a mouse model resulted in lung tumors with high penetrance for metastases [169]. Expression profiling of these tumors and human lung cancer cell lines identified a variety of metastases-related genes (NEDD9, VEGFC, CD24) as targets of LKB1. Integrated analysis of transcriptome and phosphoproteome identified the SRC kinase activation as a frequent event in tumors with LKB1 mutations [170].

Mutant LKB1 confers a major metabolic deficiency on tumors driven by Kras in genetically engineered mouse models. Dtymk, encoding deoxythymidylate kinase, which catalyzes dTTP biosynthesis, is synthetically lethal with Lkb1 deficiency in mouse and human lung cancer lines. Global metabolite profiling demonstrated that Lkb1-null cells had striking decreases in multiple nucleotide metabolites as compared to Lkb1-wt cells. DTYMK is therefore a potential therapeutic target in NSCLC [171].

Adenocarcinoma cells with mutant KRAS and loss of LKB1 are also addicted to the mitochondrial metabolism driven by lysosomal degradation of macromolecules which, in turn, depends on coatomer complex 1 (COP-1) [172].

##### Potential treatment options for LKB1-deficient cancers

In a mouse model mentioned above, triple inhibition of SRC, MEK1, and PI3K resulted in tumor regression [170]. Biguanide compounds traditionally used for treatment of diabetes have shown activity in pre-clinical models of NSCLC with LKB1 and KRAS mutations [173]. In this study, metformin, in wide use for diabetes, was less potent than phenformin, which fell out of favor because of its tendency to induce lactic acidosis in some patients. Neither is likely to be effective as single treatments for NSCLC with LKB1 mutations. Metformin is currently being tested in combination with Paclitaxel, Carboplatin, and Bevacizumab in a phase 2 randomized clinical trial NCT01578551 for adenocarcinoma of lung.

#### 1.6 TP53 PATHWAY

##### TP53

Inactivating mutations in TP53 are the most common alterations affecting any specific gene in human cancer. Inactivating mutations in TP53 disrupt its key function in controlling cellular proliferation and apoptosis. Some mutations in TP53 confer “gain of function”, and the resulting mutant TP53 protein actually contributes to different stages of tumorigenesis or to the drug resistance. Mutations in TP53 in lung cancer are strongly associated with smoking, and are also the most common somatogenic alterations in NSCLC, occurring in over half of AC, 80% of SQCC and 70% of SCLC. The TP53 locus is also frequently affected by copy losses. Mutations in TP53 are associated with poor prognosis [174] and resistance to chemotherapy.

##### MDM2

E3 ubiquitin ligase MDM2 binds to TP53 and promotes degradation of p53 through ubiquitin-proteasome pathway. MDM2 is amplified in 22% of NSCLC patients with and is associated with poor OS [175]. Prevention of MDM2 binding could restore TP53 function in wild-type TP53 tumors.

The MDM2-TP53 interaction has attracted serious efforts to develop specific inhibitors. Discovery of Nutlin-3, a promising inhibitor of MDM2 binding [176] energized the search for clinically active drugs. Early clinical testing of one such inhibitor, JNJ-26854165, did not provide a basis for continuation. The new generations of Nutlin-based and other drugs are in clinical studies for various solid tumors. Encouraging results were reported for one of them, R7112/RO5045337, in early clinical testing for liposarcoma and other tumors [177]. Other drugs – SAR405838 and RO5503781, also small-molecule inhibitors of the MDM2-TP53 interaction – are in clinical development for cancers other than lung. APR-246 (PRIMA-1MET), a drug that restores activity of unfolded or mutant p53, was safe and showed indications of clinical activity in hematological malignancies and prostate cancer [178].

#### 1.7 RB1 PATHWAY

##### RB1

RB1 is a well-known tumor suppressor with an important role in controlling the cell cycle, that is frequently inactivated in many human cancers. Hypophosphorylated RB controls the transcription factors E2F1, E2F2, and E2F3, which are necessary for the G1 to S transition of the cell cycle. In normal cells, RB1 is phosphorylated by the cyclin D/CDK4 complex to release E2F factors for initiation of the cell cycle. Mutations of RB1 are rare in AC, more frequent in SQCC (7%), and ubiquitous in SCLC at over 90% of tumors [179]. Nevertheless, frequent disruption of the CDKNA2 locus in SQCC significantly increases the involvement of RB1-mediated pathways in this type of lung cancer.

##### CDKNA2 locus

Two tumor suppressors are encoded within this CDKNA2 locus: p14ARF, which activates p53 through inhibition of its major negative regulator MDM2; and p16INK4a, a cyclin-dependent kinase inhibitor that activates retinoblastoma (RB) through negative regulation of CDK4. Loss of CDKN2A was reported to occur in 15% of SQCC and in >20% of adenocarcinoma. P16INK4a is made nonfunctional by other types of alterations in SQCC, such as epigenetic silencing, exon skipping, and mutations, bringing the percentage of SQCC tumors with dysfunctional p16INK4a to 72% [4].

##### Potential therapeutic approaches to alterations in RB pathway

Potential treatment of p16INK4A deficiency could involve inhibition of CDK4/6. A trial of the CDK4/6 inhibitor PD0332991 in selected patients with advanced NSCLC and disrupted p16INK4a is ongoing (NCT01291017). BAY1000394, a pan-CDK inhibitor, is currently being tested in a phase 1/2 trial in SCLC (NCT01573338), in combination with chemotherapeutic drugs (no placebo arm).

#### 1.8 MYC PATHWAY

##### MYC family

The protoocogene and master regulator of transcription, MYC, controls transcription networks governing proliferation, metabolism, ribosome biogenesis, and protein synthesis. However, when overexpressed, MYC induces expression of pro-apoptotic protein BAX, which induces mitochondrially mediated apoptosis. MYC is amplified in >30% of adenocarcinomas and in 16% to 30% of SCLC patients. MYC-family genes such as MYC, MYCL1, and MYCN are amplified in a mutually exclusive manner in SCLC, as observed in other tumors of neuroendocrine origin.

Experiments in a mouse model demonstrated that MYC is a valid therapeutic target in KRAS-driven NSCLC whose systemic inhibition eradicates tumors [180]. However, MYC is a notoriously difficult therapeutic target, both because of its dominant role in many normal cellular processes, and because the tertiary structure of MYC protein is challenging in respect to modeling of inhibitors. Nevertheless, the possibility of targeting downstream effectors of MYC remains viable. Thus, SCLC with MYC amplification is sensitive to inhibition of aurora kinase B (AURKB) [181].

The anti-apoptotic protein MCL1 is overexpressed and amplified in over 30% of AC [2, 182]. Examination of MCL1 in NSCLC patients showed no prognostic value [183]. However, subsequently MCL1 was identified as a protein whose expression is involved in survival of tumors that have high levels of the oncogene MYC. The majority of MYC- overexpressing NSCLC were positive for expression of MCL1, and together expression of these two genes significantly correlated with poor prognosis [184]. MCL1 is a member of the BCL2 family of anti-apoptotic proteins; inhibitors of this family have already been tested in men, and are still in clinical trials.

Transcriptional activity of MYC might be one of the converging factors of the activation of MAPK and PI3K pathways, and it could be targeted at the level of MYC accessory transcription factors such as bromodomain-containing proteins [185]. A BET bromodomain inhibitor, JQ1, showed remarkable antitumor activity in a mouse model of KRAS-driven NSCLC, albeit sensitivity was abrogated in tumors containing both KRAS mutation and inactivation of LKB1 [186]. Downregulation of MYC protein was key to tumor response to JQ1 treatment.

SCLC cell line tumors without amplified MYC genes show recurrent inactivation of MAX, a MYC-associated protein. MAX mediates transactivation of pro-differentiation genes by virtue of suppressing activity of MYC, and it suppresses MYC-mediated activation of stemness-related genes [187], acting as a bona-fide tumor suppressor.

##### Potential therapeutic approaches for MYC overexpression

The aurora A kinase inhibitor MLN8237/alisertib is in clinical trial phase 1/2 for lung and other cancers (NCT01045421) and, in combination with erlotinib, for NSCLC (NCT01471964). Other aurora kinase inhibitors have been clinically tested in a variety of malignancies, but most showed little promise in terms of safety and/or efficacy.

#### 1.9 EPIGENETIC PATHWAYS

Deregulation of the epigenome is now recognized to be a common feature of cancers. Epigenetics encompasses several processes, such as DNA methylation, histone modifications and RNA interference. The importance of DNA methylation, for example, was recognized when a study demonstrated clinical utility for prognostic purposes of the methylation status of a four-gene panel in early-stage NSCLC [188]. Since then, and largely due to unbiased NGS efforts, somatic alterations were identified in a number of genes involved in epigenetic regulation in all major types of lung cancer (Tables 1 and 2). Interestingly, there is no overlap between the genes involved in regulation of epigenome between three major types of lung cancer. This suggests different clinicopathological origins of the three major lung cancer types.

In AC, mutations were found in tumor suppressors SMARCA4/BRG1 (SWI/SNF-related, matrix-associated, actin-dependent regulators of chromatin) and ARID1A (AT-rich interaction domain 1A) – both members of the SWI/SNF complex, in 8% and 10% of tumors, respectively [2]. BRG1 is inactivated in lung cancer in a manner mutually exclusive with MYC amplification [189] and inactivation of both BRG1 and MAX is a synthetic lethal interaction [187]. SWI/SNF-chromatin-remodeling complex might protect cells against DNA damage by ensuring DNA repair through cell cycle arrest and apoptosis. A number of cancer-related proteins such as p21CIP, BRCA1, MYC, FOS, and LKB1 are known to associate with the SWI/SNF complex.

**SETD2** is a trimethyltransferase responsible for methylation of histone H3 on Lys36 (H3K36me3). This epigenomic histone mark ensures efficient DNA mismatch repair during replication process [190]. It is mutated in 4% to 5% of AC.

**MLL2** is a histone methyltransferase mutated in 19% of SQCC [4]. So far, this is the only gene product involved in epigenetic regulation that was found to be mutated in SQCC. MLL genes are also mutated in 10% of SCLC [5].

**CREBBP/CBP** and **EP300**, histone acetyltransferase coactivators, are mutated in 18% of SCLC [5]. Both are transcriptional coactivators with roles in many transcriptional programs and biological processes, conducted through acetylation of lysine residues on histones.

**EZH2** (enhancer of zeste homolog 2) is a methyltransferase and a component of repressive-PRC2 complex that triggers transcriptional repression by catalyzing the addition of methyl groups onto lysine 27 of histone H3 (H3K27me2/3). EZH2 expression is controlled by E2F transcription factor that are, in turn, controlled by RB1, whose loss of function is a hallmark of SCLC. EZH2 is an accepted oncogene in metastatic prostate cancer, and its expression is highly elevated in SCLC [6] where it is likely to activate a transcriptional program promoting the aggressive character of SCLC. Higher EZH2 expression in lung AC is associated with shortened recurrence-free survival and overall survival [191].

**KDM2A**, histone H3 lysine 36 demethylase, is overexpressed in a subset of NSCLC patients and is indispensable for tumorigenicity and invasiveness of KDM2A-overexpressing NSCLC cells. The primary target of KDM2A-mediated repression is the dual-specificity phosphatase 3 (DUSP3) gene, whose protein dephosphorylates ERK1/2. High KDM2A levels are associated with low DUSP3, high phospho-ERK levels, and poor prognosis [192].

Therapeutic options for targeting epigenomic alterations are limited at this time to drugs that show little or no selectivity. DNA methylation inhibitors, typically nucleoside analogs, target DNA methyltransferases, leading to demethylation of DNA and gene re-expression. Several nucleoside analogues – 5-azacytidine and 5-aza, 2′-deoxycytidine – were found to be effective in the treatment of myelodysplastic syndromes and leukemias and are FDA approved for these indications. Histone deacetylase (HDAC) inhibitors targeting acetyltransferases expand the repertoire of possible target genes, and several HDAC inhibitors have been evaluated in clinical trials with encouraging findings. Large-scale analysis of transcriptomes in AC versus SQCC tumors identified disruption of numerous histone-modifying enzymes in SQCC, and indicated that HDAC inhibition is a promising treatment strategy [193]. Phase 1/2 trial NCT00907179 addresses the safety and efficacy of Panobinostat/LBH589 in non-squamous NSCLC. NCT00387465, a phase 1/2 study, explores a combination of HDAC inhibitor entinostat/MS-275 with azacytidine in recurrent NSCLC.

#### 1.10 OXIDATIVE STRESS

##### NRF2 (NFE2L2), KEAP1, and CUL3

Nuclear factor erythroid-2 related factor 2 (NRF2 or NFE2L2) is a redox-sensitive transcription factor that positively regulates the expression of various genes with protective function against oxidative stress (antioxidants, xenobiotic, detoxification enzymes and more). Kelch-like ECH-associated protein 1 (KEAP1) is a ubiquitin ligase that negatively regulates NRF2 activity by targeting it for proteasomal degradation. Inactivation of KEAP1 is a frequent event in NSCLC [194], with mutations and copy-number losses in ~11% of AC and 12% of SQCC cases. Loss of KEAP1 function is predicted to increase signaling from NRF2, leading to an increase in anti-oxidative responses and protection of cells from cytotoxic chemo- and radiation therapies.

SQCC tumors have additional alterations in the pathway that lead to sustained signaling through NRF2, including NRF2 itself, which is mutated in 19% of SQCC tumors. Mutations are found exclusively in the KEAP1 interacting motifs [4] suggesting that they contribute to the stability rather than activation of NRF2. An additional 7% of SQCC tumors have an inactivating mutation in the CUL3 gene, which encodes an E3 ligase directly involved in degradation of NRF2. Altogether, the NRF2 inactivation is negatively affected in 34% of SQCC [4]. KEAP1 serves as an adaptor to CUL3 to initiate degradation of NRF2 [195]. Mutations in KEAP1 and CUL3 are mutually exclusive with mutations in NRF2 [4].

Nuclear localization of NRF2 (assumed by NRF2 protein that is not bound by KEAP1) was seen in 26% of NSCLCs; it was significantly more common in SQCCs (38%) than in adenocarcinomas (18%), and is associated with poor prognosis [196].

The involvement of the NRF2 pathway in tumorigenesis remains controversial, though mouse model studies indicate that in early stages of tumorigenesis NRF2 plays a promoting role, and its activation has been linked to resistance to chemotherapy (reviewed in [197]).

A recent study suggests that induction of oxidative stress by targeting enzyme SOD1 could be of benefit in NSCLC. SOD1 inhibitor ATN-224 increases levels of peroxide, activates p38 pathway, and decreases levels of anti-apoptotic protein Mcl1, inducing regression of tumors in a mouse model of NSCLC [198].

#### 1.11 DEVELOPMENTAL PATHWAYS

The involvement of reactivation of embryonic or stem cell transcriptional programs in lung cancer might be underestimated, since the NGS analyses are more geared towards identification of somatic alterations. A recent study of almost 300 lung tumors identified an ectopic gene-expression signature associated with highly aggressive tumors, which was an independent predictor of poor prognosis. The common molecular features were the acquisition of embryonic stem cell/germ cell gene expression profiles and the down-regulation of immune response genes [199].

Differentiation and embryonic development related genes and pathways are involved in all three major types of lung cancer (Tables 1 and 2), and the particular genes affected by mutations or other alterations seem to be almost predetermined by the respective roles of these genes in development of relevant lineages. The different spectra of developmental pathways altered in three major types of lung cancer, together with the observed differences in disregulated epigenetic pathways, is strong evidence of different origins of the tumor-initiating cells and pathology in AC, SQCC, and SCLC. Most of these gene products in altered developmental pathways are predictably transcription factors. Unfortunately, targeting these is currently not approaching clinical development.

##### NKX2.1 (TTF1, TTIF1)

**NKX2.1** is a homeobox transcription factor essential for development of the normal lung, in particular in branching morphogenesis. NKX2.1 is amplified in up to 20% of lung adenocarcinoma, but not in SQCC [4, 200, 201]. A number of targets of NKX2.1 have been identified, among them E2F3, cyclin B1, cyclin B2, c-Met, and RTK-like gene ROR1, with consequent activation of PI3K [202], a network of transcription factors, and vaccinia-related kinase VRK-1 [203].

Possible therapeutic implications of NKX2.1 amplification are activation of ROR1 (which sustains activity of c-Src), kinase activity-independent EGFR-ERBB3 association, ERBB3 phosphorylation, and consequential PI3K activation [204]. As an RTK, ROR1 could be targeted in the future. The kinase VRK-1, activated through NKX2.1 signaling, is also a potential target.

##### WNT pathway

WNT signaling is involved in pathogenesis of NSCLC but mutations in APC and β-catenin are uncommon. Still, there is evidence that inhibition of WNT pathway *in vitro* inhibits proliferation of lung cancer cells and downregulation of WNT-pathway inhibitory proteins in cell lines and tumor biopsies (reviewed in [205]). Vanctitumab/OMP18R5, an antibody targeting frizzled 7 in the WNT pathway, has entered clinical testing in NSCLC, in trial NCT01957007.

##### RUNX3

**RUNX3**, a developmentally important transcription factor and tumor suppressor is often methylated in lung cancer [206]. RUNX3 suppresses the development of lung adenoma in the setting of activated KRAS, and this effect is mediated by RUNX3-induced expression of p14ARF and p21WAF/CIP [207]. These findings provide a link between the pathways activated by mutant KRAS and the p53-p14 ARF pathways.

##### Disrupted differentiation program in SQCC

A number of genes involved in development and in squamous cell differentiation were found to be mutated in SQCC, in 44% of samples examined. The alterations include amplifications (TP63, SOX2); loss of function mutations in NOTCH1, NOTCH2, and ASCL4, and focal deletions in FOXP1 [4].

HMG box transcription factor SOX2 is a lineage-survival oncogene expressed during initiation of branching morphogenesis in lung, but enforced high expression of SOX2 prevents branching and differentiation [208]. SOX2 amplification was detected in SQCC of lung and esophagus, and is required for survival of SQCC cell lines [209]; an NGS study [4] identified SOX2 amplification in 21% of lung SQCC. Histone demethylase LSD1/KDM1 was found to be significantly elevated in Sox2-expressing SQCC, and inhibition of LSD1 with selective inhibitor CBB1007 had a cytotoxic effect on cell lines with SOX2 amplification [210]. LSD1 specifically demethylates histone H3K4. LSD1 inactivation directly impairs the Sox2-dependent transcriptional program by reducing the lineage-survival oncogene function and inhibiting Sox2-mediated repression of differentiation genes. These findings make LSD1 an epigenetic target for therapy in Sox2-expressing cancers.

A gene frequently co-amplified with SOX2 in 3q26 is PRKCα that phosphorylates SOX2 facilitating its recruitment to the promoter of Hedgehog (Hh) acyltransferase (HHAT). HHAT represents a rate-limiting step in the maturation of the Hh ligand [211]. PRKCα and SOX2 might act as oncogenic co-drivers in SQCC, as neither alone is sufficient for tumor initiation. PKCι-**SOX2-HHAT** axis contributes to the “stem-like” phenotype of SQCC and could be targeted with **PKCα** inhibitors or with inhibitors of HHAT that are being developed [212].

**Notch** signaling is required for the cell- fate decision between ciliated and non-ciliated secretory epithelium in developing lung [213]. Expression of Notch is associated with the stem cell compartment. Protein-truncating mutations of Notch receptors were reported in a majority of cutaneous SQCC and in SQCC of lung, but activating mutations of NOTCH1 commonly occur in human lymphoid malignancies. However, in progenitor basal cells of airway epithelium Notch signaling is required for differentiation but not cell renewal [214], perhaps explaining the role of abrogated Notch signaling in pathogenesis of lung SQCC. Inactivation of Notch is observed in 13% of lung SCC [4]. Notch signaling is active in “tumor propagating”, i.e., cancer stem cells in NSCLC models, and Notch3, in particular, is nonredundant in KRAS-driven tumors [215]. Inhibitors of β-secretase RO4929097, a protease needed for activation of Notch signaling, are in clinical trials for NSCLC, including phase 2 NCT01193868, and in combination with erlotinib in NCT01193881 and with cediranib NCT01131234.

**TP63** is considered to be a marker of stem cell compartment in different lineages, and is a regulator of squamous cell differentiation involved in the development of tracheobronchial epithelium [216]. Detection of TP63 by immunostaining is a useful marker that distinguishes lung SQCC from SCLC [217]. Amplification of TP63 was reported in SQCC-derived cell lines [218] and in 16% of tumors [4]. An *in vivo* model of SQCC (not of lung origin) showed that TP63 signaling is essential for tumor growth, and identified a survival program governed by TP63 that involves signaling from FGFR2 activated by stroma-derived ligand [219]. Clinical FGFR inhibitor AZD4547 was highly efficient in ablating this TP63 induced paracrine signaling in the mouse model.

Truncations of **ASCL4**, a member of the achaete-scute complex family of genes, were found in 3% of SQCC [4], and are thought to affect a developmentally important gene based on analogy to ASCL1, a related gene product essential for survival of SCLC (see below).

##### Involvement of developmental pathways in SCLC

A limited number of development pathways are inappropriately activated in SCLC, some related to the neuroendocrine nature of this deadly cancer. Changes in expression of some key developmentally important genes are more characteristic for SCLC than are mutations.

**ASCL1** is a lineage-specific, basic helix-loop-helix member of the achaete-scute family, and it is essential for differentiation of numerous pulmonary neuroendocrine (NE) cells and tissues during lung development [220]. ASCL1 is highly expressed in SCLC [221]. Hes-1 is a transcriptional target of the Notch pathway and it blocks transcription of ASCL1. The Notch pathway is suppressed in SCLC [222], and activation of Notch signaling in SCLC induces growth arrest, suggesting that this pathway could be explored as a therapeutic target [223].

**Hedgehog (HH)** pathway is frequently activated in SCLC [224] as seen in the immunochemistry analysis of tumors [225]. Nevertheless, somatic alterations in the HH pathway have not been detected. Deletion of hedgehog- signaling-molecule Smoothened (Smo) halted development of SCLC in a mouse model [226]. Activation of HH signaling after chemotherapy appears to be critical in development of chemoresistance, which is an inherent feature of SCLC. The HH pathway is a potentially druggable target, at least in SCLC mouse models, but it is unknown what specific genetic alterations in SCLC are associated with the activation of HH signaling. One phase 1 trial for SCLC is ongoing that compares chemotherapy alone or in combination with HH inhibitor GDC-0449/vismodegib or with cixutumumab NCT00887159. New inhibitors of Hh signaling targeting enzyme HHAT are in development [212].

**SLIT** proteins are secreted ligands for ROBO receptors, which are involved in axon guidance and cell migration. Robo1-deleted mice do not develop normal lungs and most die early after birth; survivors die within the first year of life and show epithelial bronchial hyperplasia [227]. Both SLIT2 and ROBO1 were shown to act as tumor suppressors in lung cancer [228, 229]. SLIT2 is mutated in 10% of SCLC patients [5].

**EPHA7** (ephrin receptor A7) is frequently lost in lymphomas and other tumors and acts as a soluble tumor suppressor. EPHA7 plays an important role in development and regulates repulsion versus adhesion of migrating cells, including during neural tube formation [230]. EPHA7 is mutated in 10% of SCLC [5].

##### Repositioning of antidepressants for treatment of SCLC

The neuroendocrine nature of cells comprising SCLC tumors might offer unorthodox treatment options. A recent study has used a bioinformatics approach to identify drugs of potential benefit in treating SCLC. A no-longer-used class of antidepressants, tricyclics, was found to have a potent cytotoxic activity in SCLC *in vitro* and *in vivo*. Tricyclics disrupt the autocrine survival signals involving neurotransmitters and their G protein–coupled receptors [231]. On the basis of these findings, phase 2a clinical trial NCT01719861 examining tricyclic desipramine in SCLC is recruiting patients.

#### 1.12 OTHER RECURRENT ALTERATIONS

The gene encoding the catalytic subunit of telomerase (TERT) shows copy gains in 42% of AC [2]. The telomerase inhibitor imetelstat sodium/GRN163L (antisense oligonucleotide) is currently being trialed in breast cancer, and a telomerase vaccine GV1001 is being trialed in NSCLC (NCT01579188).

Even though a number of studies reported an association between certain polymorphisms in DNA-damage repair genes and incidence of lung cancer, large-scale studies have not revealed recurrent mutations in this group, except for ATM in AC [2]. ATM shows in-frame indels and truncating mutations in ~10% of AC [2].

SCLC cells show increased levels of PARP-1 expression compared with NSCLC cells, and are significantly more sensitive to PARP-1 inhibition, suggesting that PARP-1 is a valid therapeutic target in SCLC [6]. In particular, a new PARP-1 inhibitor, BMN-673, is highly active against SCLC *in vitro* and *in vivo*, but PI3K pathway activation is associated with resistance to BMN-673 [232]. PARP-1 inhibitors are in clinical testing for SCLC, either as a single agent (BMN 673 in phase 1 trial NCT01286987), or in combination with temozolomide (veliparib in phase 2 trial NCT01638546).

### 2 IMMUNOTHERAPY FOR LUNG CANCER

Immunotherapy has become an exciting area in oncology, with new therapies directed at various tumors, with some early successes in particular with immunomodulatory antibodies. More clinical research is now being conducted in NSCLC. Lung cancer in general is not known as an immunogenic cancer, with very few articles addressing immune infiltrates in lung tumors. This is very much unlike the well-characterized presence of infiltrates in melanoma and other cancers, where they are known to have a proven predictive significance. Nevertheless, early successes with immunomodulatory antibodies in NSCLC have been reported (see below and review [233]). It is already apparent that responses to immunotherapy, though shown to be rare in current studies, tend to be long lasting, unlike what is seen with most targeted therapies.

Experience with small-molecule targeted therapies across cancer types has revealed a relatively high rate of response of limited duration followed by development of resistance. Conversely, immunotherapy agents tend to produce responses in fairly small subpopulations of patients but these responses are often durable. The biggest challenge in developing successful immunotherapies is the identification of biomarkers predictive of response. The incorporation of immunotherapy markers into clinical studies is of high importance, which has been demonstrated in a trial of the anti-PD-1 antibody in several cancers [234]. Expression of the PD-1 ligand (PD-L1) was shown to be a very strong predictor of response in this study. Unfortunately, testing for predictive biomarkers of immune response is still in its infancy and is not always conducted even if a target of intervention is known (such as expression of PD-L1). Similarly, a vaccination trial could be conducted with a defined tumor-associated antigen, but its expression in a given patient's tumor is not routinely examined.

In addition, the accepted frameworks for evaluation of the anti-cancer drug efficacy, Response Evaluation Criteria in Solid Tumors (RECIST), and WHO criteria, might not be appropriate for the evaluation of immunotherapeutic agents, because the latter might produce responses that are heterogeneous and could be delayed or even involve an early increase in tumor burden. Therefore, assessment of responses to immune therapy is being reconsidered [235].

In general, immunotherapeutic approaches in lung cancer might have to be multi-pronged, involving both stimulation of immune responses and inhibition of immune checkpoints, or combination with chemo- or targeted therapies that might, in certain scenarios, increase the efficacy of immunotherapies.

#### 2.1 Blockade of immune checkpoints

Inhibition of immune checkpoints must rely on presence and functional activity of the tumor infiltrating cells (TIL). Even though immunomodulatory antibodies targeting CTLA4 or PD-1 interactions with its ligands showed promising responses in lung cancer, the non-responsive patients might benefit from additional stimulation of TILs and /or modulation of tumor microenvironment that dampens immune responses (reviewed in [236]. The role of oncogenic pathways in orchestrating immune escape is exemplified by the upregulation of PD-L1 in NSCLC with mutant EGFR [7]. Inhibition of EGFR enhanced T cell function and improved survival of tumor-bearing mice [7]. A recent study conducted in animal model (breast cancer) suggested that irradiation leads to elevated expression of PD-L1 in tumors and increases the efficacy of anti-PD-L1 treatments [237]. Observations like this may be useful in mapping new strategies for co-treatments with immune checkpoint antibodies.

**CTLA4** is an inhibitory molecule expressed on T cells that is involved in the negative regulation of T cell interaction with antigen-presenting dendritic cells (APCs). CTLA4 inhibits binding of CD28 on T cells to B7 proteins on APCs, thus weakening the costimulation of T cells. CTLA4 is also expressed on tumor cells [238]. Available results from clinical trials indicate that the response rates to CTLA4 blockade with human monoclonal antibodies ipilimumab and tremelimumab are at most 18%, but responses tend to be more durable than those seen with cytotoxic therapies. Significant autoimmune toxicities were reported in a number of trials, and, interestingly, strong association of immune-related toxicities and responses were observed [239].

A recent phase 2 study of chemotherapy and ipilimumab in advanced NSCLC showed a statistically significant improvement in PFS in patients initially treated with carboplatin and paclitaxel for two cycles; they then received ipilimumab with chemotherapy for four more cycles [240]. Overall survival was 12.2 months, similar to results from the combination of carboplatin, paclitaxel, and bevacizumab. Patients with squamous histology showed better response compared to those with nonsquamous histology. Given these results, a current phase 3 study (NCT01285609) will randomize 920 patients with SQCC to phased-in ipilimumab with carboplatin and paclitaxel versus chemotherapy alone. A similarly designed phase 3 trial (NCT01450761) compares ipilimumab to placebo in SCLC patients receiving platinum and etoposide. Phase 2 trial NCT01331525 addresses the same combination as one-arm study for patients with extensive-stage SCLC.

Phase 1 trial NCT01820754 is currently evaluating the addition of ipilimumab to standard treatment for patients with previously untreated NSCLC. Phase 2 trial NCT01471197 is comparing ipilimumab to pemetrexed in non-squamous NSCLC.

Ipilimumab is also being evaluated in combination with anti-KIR antibody BMS-986015 in patients with NSCKC, CRPC and melanoma in the phase 1 trial NCT01750580. BMS-986015 targets killer-cell immunoglobulin-like receptors (KIR) expressed on natural killer (NK) cells, therefore blocking the inhibitory function of KIR.

##### PD-1

PD-1 plays a major role in the tumor immune escape by inhibiting survival, proliferation, and immune function of T cells through interaction with its ligands PD-L1 and L2. Unlike CTLA-4, expression of PD-1 is not limited to T cells and is found in B cells and some myeloid cells. PD-1 ligands, PD-L1 and PD-L2, have different expression patterns, with PD-L1 found on multiple normal and cancerous cells including lung tumors, where it could provide, once bound by PD-1, peripheral tolerance to “self” antigens. PD-L2 is expressed on APC cells, providing tolerance to orally administered antigens. Interactions between PD-1 and its ligands attenuate immune responses [241], and, in the context of cancer, serve to protect tumor cells from cytotoxic T cells.

Completed clinical studies of humanized antibodies directed against PD-1 and PD-L1 showed a good safety profile and remarkable antitumor activity in subsets of patients with metastatic disease, including lung cancer, which is considered to be a tumor type not responsive to immunotherapy. Future development of the PD-1 pathway blockade will have to include identification of predictive biomarkers of response [236].

In a trial for several different malignancies, anti-PD1 antibodies were well tolerated and the maximum tolerated dose has not been reached [234]. This study found a strong correlation between pretreatment tumor expression of PD-L1 and responses. The better safety profile of PD-1 antibody versus CTLA-4 antibody is most likely due to the fact that the latter targets a peripheral interaction between T cells and APCs. Inhibition of PD-1 probably affects peripheral interactions as well, through PD-L2 on APCs, but it might act more locally at tumor sites through blocking the interaction of PD-L1 on tumor cells with PD-1 in tumor-infiltrating T cells. Anti-PD-L1 antibody was also tested in several cancers, including melanoma, and objective responses were observed in 9 of 52 melanoma patients; the responses were durable [242].

Nivolumab (MDX-1106, BMS-936558) is a fully human monoclonal immunoglobulin (Ig)G4 antibody that binds PD-1 with high affinity and blocks its interaction with both PD-L1 and PD-L2. A phase 1 trial showed limited responses in patients with different malignancies, including mixed responses in one enrolled NSCLC patient [243]. In a phase 2 trial, nivolumab showed long-lasting, objective responses (OR) in patients with solid tumors – including NSCLC patients, of whom 14 of 76 responded [234]. Responses were dose-dependent in NSCLC, but not in melanoma or metastatic renal cell carcinoma patients enrolled in the trial. Expression of PD-L1 on available pretreatment biopsies was a predictive marker of response to Nivolumab. A response rate of 36% was observed in patients with PD-L1 positive tumors, and 0% in the PD-L1-negative group [234].

Nivolumab is currently in a number of trials in patients with NSCLC and two trials specifically for SQCC, either as a single agent or in combination with other targeted therapies or chemotherapy agents or ipilimumab NCT01454102 Combination of nivolumab and ipilimumab has already produced outstanding responses in patients with metastatic melanoma [244], and now entered trials for other cancers, including NSCLC NCT01928394. Some of these trials include testing for PD-L1 expression in tumors, which have been confirmed to be predictive of responses to nivolumab (J Clin Oncol 31, 2013 (suppl; abstr 3016)). Nivolumab is also being tested in a phase 1 trial (NCT01714739) in combination with anti-KIR antibody BMS-986015 and in NCT01629758 in combination with IL-21. The role of demethylating agents in modulating the response to immune-checkpoint inhibition is explored in trial NCT01928576; this involves pretreatment with azacytidine, entinostat, or CC-486 which, according to a recent study might sensitize NSCLC to anti-PD1 therapy [245].

Another anti-PD-1 antibody, MK-3475 (Lambrolizumab), produced lasting responses in previously treated NSCLC patients at the rate of 24%. Median progression-free survival has not been reached and will be at least 52 weeks (15th World Conference on Lung Cancer, 2013). MK-3475 is currently in clinical trials for NSCLC, including the phase ½ trial NCT01840579 that will determine the MTD first, after which Lambrolizumab will be tested in combination with chemotherapy. The phase 1 trial NCT01295827 will test several doses of the antibody in patients with NSCLC and other cancers, and will also examine expression of PD-L1 in tumors, based on the results cited above that showed a strong correlation of the efficacy of Nivolumab with PD-L1 expression. MK-3475 is also tested in phase 2/3 study NCT01905657 for patients whose tumors express PD-L1.

##### PD-L1

BMS-936559, a high-affinity human IgG4 anti-PD-L1 that blocks PD-L1 binding to PD-1, showed a promising clinical activity and good safety profile in a phase 1 trial, with objective responses observed in 5 of 49 NSCLC patients enrolled [242]. This multicenter study, NCT00729664, is ongoing.

Other anti-PD-L1 antibodies are also in clinical development. MPDL3280A/RG7446 is currently being tested as a single-agent in two clinical trials: NCT01846416, a phase 2 trial for locally advanced or metastatic NSCLC, and NCT01375842, a phase 1 trial examining several different malignancies including lung cancer. Preliminary results from the former trial found that PD-L1 tumor expression and T cell gene signature correlate with response to MPDL3280A. MPDL3280A therapy led to T-cell reactivation and restored antitumor immunity (J Clin Oncol 31, 2013 (suppl; abstr 3001)). The phase 1 trial NCT01633970 is currently evaluating the combination of MDLP3280A with bevacizumab (Avastin) and chemotherapy in patients with advanced cancers. A recent report (European Cancer Congress 2013, Abs. 3048) announced that a significantly higher response rate to MPDL3280A was observed in smokers versus patients that never smoked (26% versus 10%).

Another anti-PD-L1 antibody, MEDI4736, entered early clinical testing recently in phase 1 trial NCT01693562 and phase 1 NCT02000947 in combination with tremelimumab (anti-CTLA-4 antobody).

##### Other immune therapies in development

**Urelumab** is a humanized agonistic monoclonal antibody targeting the CD137 receptor, a member of the TNF family of receptors, also known as 4-1BB. It has potential immunostimulatory and antineoplastic activities. Urelumab specifically binds to and activates CD137-expressing immune cells, stimulating an immune response – in particular, a cytotoxic T cell response – against tumor cells. An agonistic antibody, BMS-663513, was in clinical trials for different tumors. Safety concerns caused suspension of the trial after several patients developed liver problems, including high-grade hepatitis. However, the antibody has a promise in expanding the repertoire of functional CD8+ effector cells during T cell expansion for autologous cell transfer (ACT).

**GITR** is a costimulatory receptor expressed after T cell activation that enhances T cell function and survival. Importantly, GITR also negatively affects regulatory T cells (Tregs), and treatment with GITR agonistic antibody destabilizes intra-tumor Tregs allowing for more efficient cytolysis by CD8+ T cells [246]. A trial with anti-GITR antibody TRX-518 is ongoing in melanoma patients.

**OX40** is not involved in effector T cell activation, but rather, promotes T cell survival and expansion. In a clinical study, based at the Portland Providence Medical Center in Oregon, patients received three infusions of the agonistic mouse anti-OX40 antibody within a week. The nature of the antibody precluded further treatments. Nine of 27 patients experienced minor tumor shrinkage, although none met RECIST (response evaluation criteria in solid tumors) criteria for objective responses.

**CD40**. Unlike the costimulatory targets above, CD40 is expressed on APCs, while its ligand is expressed on T cells. Binding of the two acts as a powerful enhancer of APCs' ability to present antigens and activate T cells against foreign targets. A large number of cancer patients received infusions of agonistic antibody CP870,893 and some responses were observed [247]. A surprising finding was that treatments did not increase numbers of TILs in the tumors. In a mouse model, antibody treatments induced an influx of macrophages into tumors, presumably with enhanced cytotoxic activities.

#### 2.2 Vaccine interventions

Vaccine therapy has been studied for decades in lung cancer, even though it is unlikely that stimulation (even specific) of the immune system alone could be sufficient to block growth of lung cancers. The concept is that vaccines, made from proteins drawn from tumor cells or from oncolytic viruses, can trigger the immune system to develop memory and result in long-lasting responses to present or future tumors. Several vaccines based on different antigens are in trials for lung cancer, including some in phase 3 [248, 249]. Antigen-specific vaccines that are being clinically tested include melanoma-associated antigen-A3, liposomal BLP-25, TG4010 (MUC-1 based), and recombinant human epidermal growth factor (EGF). Other vaccines are cell-based, such as belagenpumatucel-L.

**MAGE-A3** is an antigen present on about 35% of patients with NSCLC, and thus has been chosen as a antigen in several vaccination studies. As is often the case in immunotherapy trials, this strategy has shown few but lasting responders [250]. A phase 3 trial, NCT00480025, is ongoing for patients who have undergone tumor resection. A gene- expression signature associated with response to MAGE-A3 vaccination has been identified. The set of genes whose expression was predictive of response contained mainly immune-related transcripts such as in the interferon-gamma pathways and specific chemokines. This suggests that the tumor's immune microenvironment plays a major role in the patient's clinical response [251].

**L-BLP25** (Stimuvax) targets the exposed core peptide of MUC1, a heterodimeric glycoprotein overexpressed in lung cancers that is also overexpressed in a number of epithelial cancers and is associated with poor prognosis. L-BLP-25 is a liposomal formulation of vaccine targeting MUC-1. Results from early trials with L-BLP25 were encouraging, but the phase 3 trial (Stimulating Targeted Antigenic Responses To NSCLC [START]) in unresectable stage 3 patients did not meet its primary endpoint of OS. One of the problems that contributed to failure could be that patients were not stratified based on expression of MUC-1. A companion study (NCT01015443) that was being conducted in Asian patients (INSPIRE: Cancer Vaccine Study for Stage III, Unresectable, Non–small-cell Lung Cancer in the Asian Population) is not completed yet. A trial involving combination of BLP25 with Bevacizumab is ongoing for inoperable patients with stage 3-4 NSCLC after receiving chemo/radiotherapy (NCT00828009).

Trial NCT01383148 (phase 2/3) also studies MUC-1-based vaccine TG4010 developed on the backbone of vaccinia virus Ankara that was modified to express MUC-1 and IL-2.

##### rEGF

NCT01444118 is a phase 3 trial that continues assessment of a vaccine composed of humanized recombinant EGF in hope that the immune response elicited will target circulating EGF in NSCLC patients and prevent activation of EGFR on tumors.

##### Telomerase

Phase 3 trial NCT01579188 with telomerase-based vaccine GV1001 showed a significant increase in PFS in inoperable NSCLC patients who received chemo/radiotherapy first. Increased PFS was observed only in patients who developed GV1001-specific T cell memory responses and IFNγ(high)/IL-10(low)/IL-4(low) cytokine profiles. The long-term follow-up of responders from the completed phase 2 trial demonstrated increased OS with some long-term responders.

##### NY-ESO-1

The DEC-205-NY-ESO-1 fusion protein vaccine, infused directly into lymph nodes, as a single agent or in combination with sirolimus is being examined in clinical trial NCT01522820 for various malignancies including NSCLC. Eligible patients are either at high risk of recurrence or have minimal residual disease.

**HyperAcute/Lung** (tergenpumatucel-L) is a vaccine treatment consisting of genetically modified allogeneic NSCLC cells bearing alpha-(1,3)-galactosyltransferase. NewLink Genetics has completed phase 2 clinical study NCT01774578 examining tergenpumatucel-L as a single agent in previously treated NSCLC, where 8 of 28 patients had stable disease for > 16 weeks, with one patient surviving 50 months. Tergenpumatucel-L might also have a sensitizing effect on chemotherapy, since patients who received salvage chemotherapy after progressing on tergenpumatucel-L had a better overall response to treatments than patients who had not received prior tergenpumatucel-L. The trial is now recruiting patients for phase 3.

##### Indoleamine 2.3 dioxygenase (IDO)

IDO is an immune regulatory protein that suppresses activity of CD8+ cytotoxic T cells. Cancer cells and dendritic cells might produce increased levels of IDO in a variety of cancers. IDO is targeted using inhibitors in various cancers; recently a completed phase 1 vaccination trial (NCT01219348) showed long-lasting clinical benefits in almost half of patients with stage 3-4 NSCLC [252].

##### WT2725

Trial NCT01621542 examines activity of peptide vaccine WT2725 derived from Wilms tumor protein that is highly expressed in a variety of malignancies.

A pilot trial, NCT01349647, designed for patients with SCLC after first-line chemotherapy, will test KLH conjugates of GD2L, GD3L, Globo H, fucosyl GM1, and N-propionylated polysialic acid, with the adjuvant OPT-821. The moieties used in the vaccine are known to be expressed in SCLC. Patients will be monitored for immune response and levels of CTC.

**Racotumomab** is an anti-idiotype murine monoclonal antibody (MoAb) specific to P3 MoAb with anti-metastatic effect. Racotumomab binds to the idiotype region of P3 MoAb and functionally mimics the three-dimensional structure of N-glycolyl ceramides of mono-sialyl lactose, the antigenic target of P3. As a result, this anti-idiotype antibody may stimulate the host immune system to elicit humoral and cellular immune responses against tumor cells expressing NeuGc-GM3 gangliosides. A randomized phase 2 trial (NCT01240447) and a phase 3 trial (NCT01460472) are currently being conducted in Cuba for patients with advanced NSCLC concomitant with best supportive care.

##### Vaccinia-virus-based inducers

Vaccination is currently being analyzed in mesothelioma patients in a phase 2 trial (NCT01569919) with recombinant-modified vaccinia Ankara (MVA) viral vector encoding the 5T4 fetal oncoprotein (MVA-h5T4) widely expressed in mesothelioma. The vaccine will be administered prior to and concomitant with the accepted chemotherapy regimen for mesothelioma (pemetrexed and cisplatin).

**Lucanix**, or Belagenpumatucel-L, a vaccine consisting of allogeneic NSCLC cells transfected with an antisense plasmid to TGFβ2 was in clinical trials [253], with promising results, and is now in phase 3 trial NCT00676507. FANG, a new generation of cell-based vaccine, is an experimental autologous cell vaccine based on harvested tumor cells modified to express shRNA to Furin and express GM-CSF from one vector. Furin suppression is expected to inhibit immunosuppressive signaling through its targets TGFβ1 and β2. Phase 1 trial NCT01061840 testing FANG is designed for patients with inoperable solid tumors of several different histologies including lung.

##### ISCOMATRIX

Trial NCT01258868 is examining autologous tumor cell vaccination using ISCOMATRIX adjuvant in combination with celecoxib in patients undergoing surgery for lung cancer. ISCOMATRIX is a saponin-based adjuvant that could be combined with various antigens to induce antibody or T cell–based immune responses.

##### Dendritic cell vaccines

An immunotherapy protocol involving metronomic cyclophosphamide (mCTX) followed by vaccinations with tumor-antigen-loaded, dendritic-cell-derived exosomes (Dex) is in development in France. The agent mCTX inhibits Treg functions, restoring T- and NK-cell-effector functions, and combined with Dex is able to activate the innate and adaptive immunity, according to the trial site information (NCT01159288). Phase I trials showed the safety and feasibility of Dex vaccines.

Autologous dendritic cells modified with adenovirus – employed to express chemokine CCL21 involved in recruitment of T cells – are used for vaccination of previously treated stage 3-4 patients in trials NCT00601094 and NCT01574222.

##### Allogeneic cell vaccines

A randomized phase 1 trial NCT01433172 will test a vaccine consisting of the irradiated GM.CD40L bystander cells (K562 cells modified to express GM-CSF and CD40L) and an equivalent number of allogeneic tumor cells plus or minus CCL21 involved in recruitment of T cells.

## DISCUSSION

This review aimed to summarize the current understanding of molecular pathways in lung cancer, in particular as related to their potential to be therapeutically targeted. Lung cancer is in essence a collection of diverse diseases, even though different histology does not necessarily belie different molecular blueprints of tumors, as is the case with large cell carcinoma. The latter was recently shown to belong to either AC or SCLC group, based on molecular alterations present in these tumors. Molecular blueprints of tumors are emerging as definitive determinants of the course of therapy to choose, even though presumably not all driver mutations have been identified in lung cancer, and many of the identified driver mutations are currently not targetable.

Several oncogenic pathways are involved in lung cancer, but most genetic alterations (with the exception of TP53) are not highly recurrent in a majority of cases within each histological type. For example, mutations and translocations in receptor tyrosine kinases do not account for the majority of cases of adenocarcinoma. Nevertheless, the development of targeted therapies for the inhibition of the receptor tyrosine kinases has transformed diagnosis and treatment of adenocarcinoma, whereas similar advances have not yet been achieved in targeting alternative oncogenic pathways in other types of lung cancer. Tumor suppressors play a seemingly ubiquitous role in lung cancer, but most of them remain elusive targets for therapeutic intervention. Still, many of the pathways involved in the development and progression of lung cancers are currently targeted in numerous clinical trials. Immune therapies – in particular, blockade of immune checkpoints – have produced highly promising results in clinical studies, and will remain a subject of intense clinical investigations. It is anticipated that investigational therapies and rational combinations thereof will lead to major advances in improving outcomes for patients with lung cancer.

Already the development of targeted and immune therapies has transformed diagnosis and treatment of lung cancer. Molecular testing for EGFR, EML4-ALK, and KRAS has become a routine diagnostic procedure in AC. Other forms of lung cancer lag behind in terms of development of targeted therapies, but with many clinical trials ongoing in SCC and SCLC this is bound to change.

Genomic testing for personalized treatment of lung cancer is now associated with improved survival, likely due to getting targeted kinase inhibitors to the right patients [1]. Patients whose tumors were genotyped had 28% better overall survival odds than those who couldn't get a molecular diagnosis for various reasons. Table 3 contains a list of drugs and their molecular targets that are currently in clinical testing. If even a portion of these drugs shows efficacy, much more effective management of patients based on molecular profiles of their cancers could be achieved.

Rapid advances in the field of targeted therapies in cancer treatment might require an overhaul of the clinical trial system. A recent example is the “Master Protocol”, a new trial design resulting from a collaboration between a genomic testing company, federal health and regulatory agencies, pharmaceutical companies, multiple cooperative groups and patient advocacy organizations. The Master Protocol is a biomarker-driven, multi-drug, multi-arm, multi-center phase 2/3–registration trial, in which a comprehensive genomic testing platform will be used for all patients to determine stratification into appropriate target-specific arms of the trial. The protocol will enroll patients with SQCC, and could be presumably adopted for other types of cancer. The use of comprehensive genomic testing rather than piecemeal testing for one mutation at a time, and the availability in the Master Protocol of multiple targeted drugs should greatly increase the probability of patients to be really matched to a targeted therapy.

The problem with targeted therapies is that most of the time clinical responses to them are short-lived. The mechanisms of resistance are multifaceted, and might involve new resistance-conferring mutations in the target protein itself (such as T790M in EGFR) or activate lateral signal-transduction pathways via new mutations or changes in the expression level of the key proteins. The first type of resistance (“gatekeeper” mutations) is currently approached by development of more potent or irreversible inhibitors (such as CO 1686 and other investigational drugs for EGFR-T790M). The second type of resistance is addressed by simultaneous targeting of two or even more oncogenic proteins or pathways.

Immune therapies, in particular immune checkpoint blockade, have shown a tremendous promise in clinical trials for lung cancer. The responses to these tend to be durable, but the problem is the heterogeneity of responses from patient to patient. A lot of effort will be expended, undoubtedly, into understanding the mechanisms that contribute to durable responses in some patients and lack of the responses in other patients with the same histological tumor subtype. It is very likely that tumor microenvironment and a particular genetic blueprint play a major role in modulation of immune responses, which might necessitate combination treatments involving targeted and immune therapies.

Development and advancement of new treatments in lung cancer will depend on the widespread introduction of comprehensive molecular diagnostic procedures and encouraging patient enrollment in clinical trials; also on reporting and comparing outcomes. In order to address clinically the ever-important problem of inherent and acquired resistance to targeted therapies, genomic analyses of resistance are of utmost importance. The success of immunotherapeutic approaches will depend on a better understanding of the basic biology of immune responses and, in particular, the role that tumor microenvironment plays in shaping immune responses.
